# Interventions for improving adherence to treatment for latent tuberculosis infection: a systematic review

**DOI:** 10.1186/s12879-016-1549-4

**Published:** 2016-06-08

**Authors:** Anke L. Stuurman, Marije Vonk Noordegraaf-Schouten, Femke van Kessel, Anouk M. Oordt-Speets, Andreas Sandgren, Marieke J. van der Werf

**Affiliations:** Pallas health research and consultancy BV, Rotterdam, The Netherlands; European Centre for Disease Prevention and Control (ECDC), Tomtebodavägen 11a, Solna, 171 65 Sweden

**Keywords:** Tuberculosis, Latent tuberculosis, Treatment initiation, Treatment adherence, Treatment completion, Risk groups

## Abstract

**Background:**

Latent tuberculosis infection (LTBI) control relies on high initiation and completion rates of preventive treatment to preclude progression to tuberculosis disease. Specific interventions may improve initiation and completion rates. The objective was to systematically review data on determinants of initiation, adherence and completion of LTBI treatment, and on interventions to improve initiation and completion.

**Methods:**

A systematic review of the literature (PubMed, Embase) published up to February 2014 was performed. Relevant prospective intervention studies were assessed using GRADE.

**Results:**

Sixty-two articles reporting on determinants of treatment initiation and completion were included and 23 articles on interventions. Determinants of LTBI treatment completion include shorter treatment regimen and directly observed treatment (DOT, positive association), adverse events and alcohol use (negative association), and specific populations with LTBI (both positive and negative associations). A positive effect on completion was noted in intervention studies that used short regimens and social interventions; mixed results were found for intervention studies that used DOT or incentives.

**Conclusion:**

LTBI treatment completion can be improved by using shorter regimens and social interventions. Specific needs of the different populations with LTBI should be addressed taking into consideration the setting and condition in which the LTBI treatment programme is implemented.

**Electronic supplementary material:**

The online version of this article (doi:10.1186/s12879-016-1549-4) contains supplementary material, which is available to authorized users.

## Background

Exposure to *Mycobacterium tuberculosis* may result in latent tuberculosis infection (LTBI). LTBI can in turn progress to tuberculosis (TB) disease, especially if the immune system is compromised [1, 2]. One-third of the world population is estimated to be latently infected with *M. tuberculosis* [3]; therefore, LTBI control is an important step towards TB elimination, in addition to TB case detection and treatment [4, 5]. LTBI control consists of identifying individuals with LTBI and offering them preventive treatment. The fact that initiation and completion rates of preventive treatment are often low and differ between treatment regimens and populations with LTBI may hamper the control of LTBI.

Numerous factors can influence patients’ medication uptake, such as forgetfulness, side effects, stigma, or lack of information on treatment requirements, thereby affecting initiation, adherence and completion rates of treatment. These factors affecting patients’ medication uptake have to be considered when designing interventions to modify complex human behaviour associated with treatment adherence [6–8]. Specific examples of interventions that have been used to improve initiation, adherence, or completion of LTBI treatment include switching from regimens with longer treatment duration to regimens with shorter treatment duration [9], incentives [10], and education or counselling [7].

In order to provide European Union and European Economic Area (EU/EEA) Members States and candidate countries with guidance on programmatic LTBI control a systematic review on initiation and completion of preventive treatment was performed in a bilateral cooperation with the World Health Organization (WHO) [11]. The review served as input for the WHO guidelines on management of LTBI launched in 2015 [11], and will be used for the EU/EEA tailored guidance. The review questions were (1) What are the initiation and completion rates of different LTBI treatment regimens?; (2) What are the determinants of initiation, adherence, and completion of recommended LTBI treatment regimens in the general population and in specific populations with LTBI?; and (3) What are interventions with demonstrated efficacy to improve LTBI treatment initiation, adherence and completion in different populations?. The current article presents the results on the latter two review questions.

## Methods

A systematic literature review was performed to provide answers to the review questions described above. This review was done according to a review protocol and following the Cochrane guidelines. The details of eligibility criteria, information sources, search strategy, study selection, and data extraction are provided in the Additional files (see Additional file 1).

### Data extraction

To answer review question 2, data on determinants of initiation, adherence, and completion were extracted for individuals with LTBI from various populations with LTBI (e.g. p-values, odds ratios (ORs), risk ratios). If univariate and multivariate analyses of the same data were presented, only the data from the multivariate analysis were extracted. Results from intention-to-treat analyses were preferred; if both intention-to-treat and per-protocol results were reported in one study, only intention-to-treat results were included. Data on non-significant factors were not consistently quantified in the studies and were therefore not listed in this review.

For review question 3, data for five groups of interventions were extracted: 1. interventions with short treatment regimens; 2. interventions consisting of directly observed therapy (DOT); 3. interventions in which incentives were offered with the treatment (e.g. cash, transportation vouchers); 4. social interventions (e.g. education, adherence coaching, peer counselling, cultural interventions); and 5. other interventions (e.g. use of interferon gamma release assay (IGRAs) rather than tuberculin skin tests (TSTs)). ORs that were adjusted for factors that related to the intervention were not used.

### GRADE

As review question 2 does not deal with the effects of health interventions, risk of bias was only assessed for aspects of the individual studies and not across the evidence base. For review question 3, the quality of the total body of evidence for each outcome (initiation, adherence, and completion rates) was critically appraised using the GRADE approach (Grading of Recommendations Assessment, Development and Evaluation; http://www.gradeworkinggroup.org). Only prospective studies (i.e. randomized controlled trials (RCTs) and prospective observational studies) were appraised using GRADE. Outcomes were downgraded for imprecision when the total number of events was less than 125 for dichotomous outcomes, and if the total sample size was less than 230 for continuous outcomes (based on estimated control group event rate of 0.60 and a relative risk difference of 30 %, given α = 0.05 and β = 0.20) [12]. GRADE tables were created using standard GRADE formats and procedures (with GRADEpro [13]). Meta-analysis was performed in accordance with GRADE methodology: summary odds ratios (sORs) and 95 % confidence intervals were calculated when the outcomes were considered relatively homogeneous regarding the intervention and the population. This was done using a random effects model with the “MAInput Table” and “MAPooledEffect” functions from the MetaXL 2.1 add-in in Excel. No quality index was used. The sORs and a measure for heterogeneity (I^2^) are shown in a forest plot [14].

## Results

### Results of the review process

Results are reported in accordance with the Preferred Reporting Items for Systematic Reviews and Meta-Analyses (PRISMA) statement. A flowchart showing the number of articles identified for all review questions during the selection process is presented in in the Additional files (see Additional file 2).

Overall, 62 articles were found for review question 2, including 27 prospective studies and 35 retrospective studies (see Additional file 3). Twelve articles provided information on determinants of initiation, eight on determinants of adherence, and 51 on determinants of completion. Twenty-three articles were found for review question 3, including twenty prospective and three retrospective studies (see Additional file 4). Among the prospective studies, seven articles described interventions with short treatment regimens, four with DOT, four with incentives, eight with social interventions, and one described another type of intervention. Four prospective studies provided data for more than one outcome [15–21].

### Determinants of LTBI treatment initiation, adherence and completion

Most determinants of LTBI treatment initiation, adherence, and completion are from studies in the general population, i.e. primarily unselected patients with LTBI at clinics (Table 1). The most frequently reported determinant associated with LTBI treatment uptake in the general population was age, though the direction of the effect was inconsistent. Most determinants related to LTBI treatment completion.

**Table 1:** Overview of determinants of LTBI treatment initiation, adherence and completion in the general population diagnosed with LTBI

Determinant	Specification determinant (vs. reference group)	Number of articles			
		Positive association		Inverse association	
		P	R	P	R
Determinants of LTBI treatment initiation					
Age	Older age (vs. younger age)	–	1 [49]	–	2 [22, 26]
Gender	Men (vs. women)	–	1 [26]	–	1 [49]
Sub-population within general population with LTBI	Refugee/immigrants (vs. born in country of study)	1 [25]	1 [26]	–	–
	Immigrants born in WHO category 3 or 5 country (vs. category 1 country)^A^	1 [25]	–	–	–
	HCW (vs. no HCW)	–	–	–	2 [22, 23]
	Case contact (vs. no case contact)	1 [24]	2 [22, 23]	–	–
Education	Lower education level (vs. n.r.)	1 [24]	–	–	–
Behaviour	Alcohol use reported at baseline (vs. no alcohol use reported)	–	–	–	1 [49]
Other	Continuity of primary care by consulting a regular physician (vs. n.r.)	1 [24]	–	–	–
	Pregnant (vs. not pregnant)	–	–	–	1 [47]
	Prior incarceration (vs. n.r.)	1 [24]	–	–	–
	Fear of getting sick with TB without medicine (vs. no fear of getting sick)	1 [24]	–	–	–
	Previous BCG vaccination (vs. n.r.)	–	–	–	1 [22]
	Abnormal CXR findings consistent with previous TB (vs. n.r.)	–	1 [22]	–	–
	A non-employment reason for screening (vs. n.r.)	1 [24]	–	–	–
Determinants of LTBI treatment adherence					
Age	Older age (vs. younger age)	–	–	1 [75]	–
Ethnicity	Bicultural^D^ (vs. Hispanic or non-Hispanic)	1 [75]	–		–
Education	Higher grades in school (vs. lower grades)	1 [75]	–	–	–
Behaviour	Risk behaviours (vs. n.r.)^E^	–	–	2 [75, 76]	–
Adverse events	Some somatic complaints (vs. n.r.)	–	–	1 [76]	–
Determinants of LTBI treatment completion					
Age	Older (vs. younger)	3 [43, 58]^B, C^	4 [29, 31, 42, 44]^G^	3 [25, 77, 78]	6 [23, 28, 30, 41, 46, 79]
Gender	Male (vs. female)	–	–	–	2 [30, 44]
Ethnicity	Hispanic/Latino ethnicity (vs. Asian ethnicity)	–	–	1 [78]	
	White Hispanic (vs. black, non-Hispanic)	–	1 [30, 34, 46]	–	–
	Country of birth (i.e. Haiti, Dominican Republic, China with HK or Vietnam) (vs. other countries)	Varying results found between countries [80]			
	Asian/Pacific Islander (vs. white)	–	2 [42, 44]	–	–
	Region of origin (i.e. Latin America and Caribbean or Asia and other) (vs. USA, Canada, Europe)	–	1 [41]	–	–
	Black race (vs. n.r.)	–	–	–	1 [29]^G^
	Ethnicity (i.e. Asian, Non-Hispanic black or Hispanic (vs. non-Hispanic white)		1 [31]		
Sub-population within source population	HCW (vs. no HCW)	–	–	–	1 [23]
	Case contact (vs. no case contact)	–	1 [31]	–	1 [29]^F^
	Currently homeless (vs. not currently homeless)	–	–	–	2 [30, 32]
	PWID (vs. no PWID)	–	–	–	2 [23, 34]
	Refugees/immigrants (vs. born in country of study)	1 [27]	4 [28–31]^G^		2 [32] [33]
	Indication for LTBI treatment immunosuppression (vs. case contact)	1 [43]^C^	–	–	–
Health	History of hepatitis A, B or C (vs. no history of liver disease)	1 [77]	–	–	–
	Other medications reported at baseline (vs. none reported)	–	–	–	1 [29]^F^
	Use of concomitant medications by women (vs. no use of concomitant medication)	–	–	–	1 [49]
Behaviour	(Excess) alcohol use (vs. no alcohol use)	–	–	–	4 [29, 30, 32, 49]^F^
	Smoking (vs. non-smoking)	1 [43]^C^	–	–	–
Treatment	Treatment without H (vs. treatment with H)	1 [43]^C^	5 [31, 39–42]	–	–
	9-months H (vs. other regimens)	–	–	–	1 [23]
	Regimen choice offered (vs. no regimen choice offered)	–	1 [79]	–	–
	Twice weekly RZ (vs. daily RZ)	–	1 [81]	–	–
	DOT (vs. SAT)	–	3 [31, 44, 45]	–	–
Adverse events	Adverse events (vs. no adverse events)	–	–	–	7 [30, 33, 41, 46–49]
	Adverse events (i.e. grade 1 or 2 hepatotoxicity, grade 3 or 4 hepatotoxicity or adverse events other than hepatotoxicity) (vs. n.r.)	Conflicting results found between adverse events [51]			
Other	Not having been incarcerated within 6 months of diagnosis (vs. n.r.)	1 [25]	–	–	–
	Referral reason (i.e. correctional/rehabilitation or postpartum women) (vs. TST positive from screening)	–	–	–	1 [28]
	Risk group (i.e. contact, medical risk^H^, population risk^I^) (vs. low risk^J^)	–	1 [31]	–	–
	Cause of screening/referral (i.e. asylum seekers or contacts) (vs. anti-TNF-α candidates)	–	–	–	1 [82]
	Fear for venepuncture (vs. n.r.)	–	–	1 [83]	–
	Low TB risk perception (vs. n.r.)	–	–	1 [83]	–
	Plan to tell friends or family about LTBI diagnosis (vs. n.r.)	1 [24]	–	–	–
	Home situation (i.e. child living with no or one natural parent) (vs. living with both natural parents)	–	–	1 [27]	–
	Spanish language (vs. non-Spanish language)	–	1 [60]	–	–
	Resident in a congregate setting (vs. never or unknown)	–	–	–	1 [23]
	Missed appointment call or letter (vs. no missed appointment call)	–	–	–	1 [60]
	No medical insurance (vs. medical insurance)	–	–	–	1 [47]
	Clinic attendance before treatment (vs. clinic non-attendance before treatment)	–	1 [79]	–	–
	Presumed non-recent TB infection (vs. presumed recent TB infection)	–	–	–	1 [34]
	Public health nurse referral (vs. no public health nurse referral)	–	–	–	1 [60]

*BCG* Bacillus Calmette-Guérin; *CXR* chest radiograph; *DOT* directly observed therapy; *H* isoniazid; *HCW* healthcare worker; *HK* Hong Kong; *i.e.* id est; *LTBI* latent tuberculosis infection; *n.r.* not reported; *PWID* people who inject drug; *RZ* rifampicin and pyrazinamide; *SAT* self-administered therapy; *TB* tuberculosis; *TNF* tumor necrosis factor; *TST* tuberculin skin test; *USA* United States of America; *WHO* World Health Organisation

^A^WHO defined 5 categories of TB prevalence based on 1st (least prevalent) to 5th (most prevalent). ^B^Data analysed in individuals that underwent three QFT-GIT. ^C^Data analysed in individuals who underwent at least one serial QFT-GIT. ^D^Bicultural is defined by questions separated into the domains Hispanic and non-Hispanic, considering language use, linguistic proficiency and electronic media use. Individuals scoring high in both domains are considered bicultural. ^E^Risk behaviours: ever used alcohol, cigarettes, marijuana, been expelled or suspended from school, or been in a physical fight. ^F^Data analysed in Hispanic subjects for one study. ^G^Data analysed in non-Hispanic subjects for one study ^H^Persons with medical risk factors such as having a TST conversion within two years of a negative TST, HIV infection, untreated or partially treated prior TB, suspected TB with an abnormal chest radiograph, being younger than five years of age with a positive TST, or having a clinical condition associated with an increased risk of TB disease. ^I^persons with population risk factors such as: recent immigrants to the USA (5 years) from countries with high TB prevalence, homeless persons, residents and employees of congregate settings such as prisons and jails, and healthcare facilities. ^J^persons with low risk for developing TB disease (no case contact, no medical risk, no population risk factors)

With regards to LTBI treatment initiation, two studies found healthcare workers (vs. non healthcare workers) to be less likely to initiate treatment [22, 23] (no study found a positive association); three studies found case contacts (vs. no case contacts) [22–24], and two studies found immigrants or refugees (vs. born in country of study) [25, 26] to be more likely to initiate LTBI treatment (no study found an inverse association). With regards to LTBI treatment completion, five studies found a positive association between completion and immigrant or refugee status (vs. born in country of study) [27–31], whereas two studies found an inverse association [32, 33]. Two studies each found that currently homeless individuals [30, 32] (vs. not currently homeless) and people who injected drugs (PWID) [23, 34] (vs. people who do not inject drugs) were less likely to complete treatment (no study found a positive association). Throughout the populations, unfavourable social risk factors were associated with worse completion [23, 25, 27, 35–38] (no study found a positive association). Determinants of treatment initiation, adherence or completion within specific source populations with LTBI are presented in the Additional files (see Additional file 3), and showed large analogy with the determinants identified for the general population. Short (vs. long) treatment regimens and treatments with DOT (vs. self-administered therapy (SAT)) were found to be completed more often in the general population with LTBI in six [31, 39–43] and three [31, 44, 45] studies, respectively, (no study found an inverse association). Adverse events were inversely associated with completion in seven studies [30, 33, 41, 46–49] (no study found a positive association). Similar results were found for determinants of completion in individuals from the different populations (see Additional file 3). Females [30, 44] (vs. males) were more likely to complete treatment (no study found men were more likely to complete treatment). Additionally, alcohol use (vs. no alcohol use) was inversely associated with completion in four studies [29, 30, 32, 49] (no study found a positive association).

### Interventions to improve LTBI treatment initiation, adherence and completion

Twenty prospective studies on interventions to improve LTBI treatment initiation, adherence, and/or completion provided evidence for the following questions (Tables 2, 3, 4, 5, 6):

**Table 2:** Grading of the body of evidence for effectiveness of short versus long LTBI treatment. Question: Does short LTBI treatment result in higher initiation, adherence, or completion rates than long LTBI treatment in individuals eligible for LTBI treatment?

			Quality assessment					n/N = %^a^	Effect		Quality	Importance
No of studies (No of participants)	Design	Population Intervention	Risk of bias	Inconsistency	Indirectness	Imprecision	Other considerations	Short LTBI treatment	OR (95 % CI)^b^	Absolute (per 1000 (95 % CI))^c^		
								Long LTBI treatment				
Initiation												
0 (0)	No evidence available	–	–	–	–	–	–	–	–	–	–	Critical
Adherence												
2 (822) [21, 50]	RCT	Case contacts	Serious^d^	Not serious	Not serious	Not serious	None	344/391 = 88 % (range: 82–92 %)	1.5 (1.0–2.3)	55 (4–92)	⊕ ⊕ ⊕O Moderate	Critical
		3HR or 2RZ vs. 6H or 9H						353/431 = 82 % (range:7–86 %)				
Completion												
1 (352) [21]	RCT	Case contacts	Serious^e^	Not serious	Not serious	Not serious	None	106/153 = 69 %	0.8 (0.5–1.3)	−46 (−156-49)	⊕ ⊕ ⊕O Moderate	Critical
		2RZ vs. 6H						145/199 = 73 %				
1 (7731) [20]	RCT	Case contacts	Very serious^f^	Not serious	Not serious	Not serious	None	3273/3986 = 82 %	2.1 (1.9–2.3)	134 (119–146)	⊕ ⊕ OO Low	Critical
		3H + RPT + DOT vs. 9H + SAT						2585/3745 = 69 %				
1 (590) [38]	RCT	Immigrants	Serious^g^	Not serious	Not serious	Not serious	None	213/296 = 72 %	2.5 (1.7–3.6)	206 (125–273)	⊕ ⊕ ⊕O Moderate	Critical
		3HR vs. 6H						154/294 = 52 %				
3 (1552) [51–53]	RCT	General population	Serious^h^	Not serious	Not serious	Not serious	None	568/785 = 72 % (range: 61–91 %)	1.9 (1.1–3.5)	141 (23–241)	⊕ ⊕ ⊕O Moderate	Critical
		2RZ or 4R vs. 6H or 9H						459/767 = 60 % (range: 57–76 %)				

Bibliography: Spyridis et al. 2007 [50]; Tortajada et al. 2005 [21]; Sterling et al. 2011 [20]; Jimenez-Fuentes et al. 2013 [38]; Menzies et al. 2008 [53]; Menzies et al. 2004 [52]; Jasmer et al. 2002 [51]

*n/N* No of individuals with LTBI who initiated, or adhered to or completed treatment/total number of subjects; *CI* confidence interval; *DOT* directly observed therapy; *3H, 6H, 9H* 3, 6 or 9 months isoniazid; *3HR* 3 months isoniazid + rifampicin; *OR* odds ratio; *4R* four months rifampin; *RCT* randomised controlled trial; *RPT* rifapentine; *2RZ* 2 months rifampicin + pyrazinamide; *SAT* self-administered therapy

^a^If >1 articles, weighed pooled point estimates and 95 % CI were calculated

^b^If >1 articles, weighed pooled estimates and 95 % CI were calculated using a random effects model (without quality index)

^c^Calculated via GradePro

^d^Spyridis et al. 2007 [50]: no blinding. Tortajada et al. 2005 [21]: no blinding; use of unvalidated patient-reported outcomes (pill count and calendar annotations); early termination (due to higher toxicity in 2RZ arm, unplanned interim analysis); dissimilarities between treatment arms (more foreign-born in 2RZ); unequal number of patients in the two groups

^e^Tortajada et al. 2005 [21]: no blinding; use of unvalidated patient-reported outcomes (pill count and calendar annotations); early termination (due to higher toxicity in 2RZ arm, unplanned interim analysis); dissimilarities in treatment groups (more foreign-born in 2RZ); unequal number of patients in the two groups

^f^Sterling et al. 2011 [20]: unclear allocation concealment; no blinding; use of unvalidated patient-reported outcomes (pill count and self-report); dissimilarities between treatment arms (with respect to North American Indians, subjects enrolled in a cluster, homelessness); exposure bias (DOT only in short treatment arm)

^g^Jimenez-Fuentes et al. 2013 [38]: unclear allocation concealment; no blinding; dissimilarities between treatment arms (with respect to sex and undocumented migration status)

^h^Menzies et al. 2004 [52]: unclear allocation concealment; no blinding. Menzies et al. 2008 [53]: unclear allocation concealment; no blinding; early termination (due to lower toxicity in 4R arm, planned interim analysis). Jasmer et al. 2002 [51]: lack of allocation concealment (alternate weeks); inadequate sequence generation (alternate weeks); no blinding; unclear treatment adherence assessment; dissimilarities between treatment arms (born outside United States, age >35 years)

**Table 3:** Grading of the body of evidence for effectiveness of DOT versus SAT. Question: Does DOT result in higher initiation, adherence, or completion rates than SAT in individuals eligible for LTBI treatment?

			Quality assessment					n/N = %	Effect		Quality	Importance
No of studies (No of participants)	Design	Population treatment intervention	Risk of bias	Inconsistency	Indirectness	Imprecision	Other considerations	DOT	OR (95 % CI)	Absolute^a^ (per 1000 (95 % CI))		
								SAT				
Initiation												
0 (0)	No evidence available	–	–	–	–	–	–	–	–	–	–	Critical
Adherence												
0 (0)	No evidence available	–	–	–	–	–	–	–	*–*	–	–	Critical
Completion												
1 (199) [17]	RCT	PWID^b^ long H	Serious^c^	Not serious	Not serious	Not serious	None	79/99 = 80 %	1.1 (0.5–2.1)	15 (−137-98)	⊕ ⊕ ⊕O Moderate	Critical
		Outreach DOT vs. SAT						79/100 = 79 %				
1 (111) [16]	RCT	PWID^b^ long H	Very serious^d^	Not serious	Not serious	Serious^e^	None	49/72 = 68 %	14.5 (5.0–42)	552 (296-732)	⊕OOO Very low	Critical
		DOT + Methadone treatment vs. SAT + no incentive^f^						5/39 = 13 %				
1 (7731) [20]	RCT	Case contacts	Very serious^g^	Not serious	Not serious	Not serious	None	3273/3986 = 82 %	2.1 (1.9–2.3)	134 (119–146)	⊕ ⊕ OO Low	Critical
		DOT + 3H + RPT vs. SAT + long H						2585/3745 = 69 %				
1 (135) [54]	RCT	Immigrants long H	Serious^h^	Not serious	Not serious	Serious^e^	None	6/82 = 7.3 %	0.1 (0.04–0.3)	−342 (−239- -387)	⊕ ⊕ OO Low	Critical
		Clinic-based DOT^i^ vs. SAT daily^c^						22/53 = 41 %				

Bibliography: Chaisson et al. 2001 [17]; Batki et al. 2002 [16]; Sterling et al. 2011 [20]; Matteelli et al. 2000 [54]

*n/N* No of individuals with LTBI who initiated, or adhered to or completed treatment/total number of subjects; *CI* confidence interval; *DOT* directly observed therapy; *H, 3H* (3 months) isoniazid; *OR* odds ratio; *PWID* people who inject drugs; *RCT* randomized controlled trial; *RPT* rifapentine; *SAT* self-administered therapy

^a^Calculated via GradePro

^b^Both studies with PWID population are presented separately, since one of the studies applies DOT + an incentive as intervention

^c^Chaisson et al. 2001 [17]: unclear allocation concealment; no blinding; use of unvalidated patient-reported outcomes in SAT arm (self-report; urine tests and MEMS in a subset of patients in this study show that self-reported adherence was greatly overestimated, thereby possibly underestimating the effect of DOT)

^d^Batki et al. 2002 [16]: no blinding; use of unvalidated patient-reported outcomes in SAT arm (monthly medication pick-up); dissimilarities between treatment arms (age, Addiction Severity Index psychiatric and Beck depression inventory); exposure bias (incentive in DOT arm)

^e^total number of events <125

^f^Approximately half of the intervention group (37/72) also received substance abuse counselling

^g^Sterling et al. 2011 [20]: unclear allocation concealment; no blinding; use of unvalidated patient-reported outcomes in SAT arm (pill count and self-report); dissimilarities between treatment arms (with respect to North American Indians, subjects enrolled in a cluster, homelessness); exposure bias (short treatment in DOT arm)

^h^Matteelli et al. 2000 [54]: unclear allocation concealment; no blinding; very large loss to follow-up; unclear treatment adherence assessment in SAT arm; unequal numbers in treatment arms; early termination (due to low completion rates in DOT arm). Early termination partially accounts for the low numbers in this study, and as we already downgraded for this (serious imprecision), we decided not to downgrade for it again in the risk of bias

^i^Most likely DOT, however terminology not very clear in the methods and results sections of the article

**Table 4:** Grading of the body of evidence for the effectiveness of (monetary) incentives. Question: Does treatment supported by (monetary) incentives result in higher initiation, adherence, or completion rates than treatment not supported by incentives in individuals eligible for LTBI treatment?

			Quality assessment					n/N = %	Effect		Quality	Importance
No of studies (No of participants)	Design	Population - treatment-intervention	Risk of bias	Inconsistency	Indirectness	Imprecision	Other considerations	Incentives	OR (95 % CI)	Absolute^a^ (per 1000 (95 % CI))		
								No incentives				
Initiation												
0 (0)	No evidence available	–	–	–	–	–	–	–	–	–	–	Critical
Adherence												
0 (0)	No evidence available	***–***	–	–	–	–	–	–	–	–	–	Critical
Completion												
1 (111) [16]	RCT	PWID - long H^b^	Very serious^c^	Not serious	Not serious	Serious^d^	None	49/72 = 68 %	14.5 (5.0-42)	552 (296-732)	⊕OOO Very low	Critical
		Methadone treatment + DOT vs. no incentive + SAT^e^						5/39 = 13 %				
1 (108) [55]	RCT	PWID - long H^b^	Not serious^f^	Not serious	Not serious	Serious^d^	None	29/53 = 53 %	32.0 (7.1–145)^g^	511 (174–809)	⊕ ⊕ ⊕O Moderate	Critical
		Monetary incentive vs. no incentive						2/55 = 3.6 %				
1 (216) [15]	RCT	Inmates^h^ - long H	Not serious^i^	Not serious	Not serious	Serious^d^	None	14/113 = 12 %	1.1 (0.5–2.4)^j^	7 (−58–124)	⊕ ⊕ ⊕O Moderate	Critical
		Non-cash^k^ incentive vs. no incentive						12/103 = 12 %				
1 (119) [56]	RCT	Homeless - long H or short HR	Serious^l^	Not serious	Not serious	Serious^d^	None	58/68 = 85 %	1.7 (0.7–4.3)	80 (−69–164)	⊕ ⊕ OO Low	Critical
		Cash vs. non-cash incentive^m^						44/57 = 77 %				

Bibliography: Tulsky et al. 2004 [56]; Batki et al. 2002 [16]; Malotte et al. 2001 [55]; White et al. 2002 [15]

n/N: No of individuals with LTBI who initiated, or adhered to or completed treatment/total number of subjects; CI: confidence interval; DOT: directly observed therapy; H: isoniazid; HR: isoniazid and rifampicin; OR: odds ratio; PWID: people who inject drugs; RCT: randomised controlled trial

^a^Calculated via GradePro

^b^Both studies with PWID population are presented separately, since one of the studies applies incentive + DOT as intervention

^c^Malotte et al. 2001 [55]: unclear sequence generation; partly blinded

^d^Batki et al. 2002 [16]: no blinding; use of unvalidated patient-reported outcomes in SAT arm (monthly medication pick-up); dissimilarities between treatment arms (age, Addiction Severity Index psychiatric and Beck depression inventory); exposure bias (DOT in incentive arm)

^e^Approximately half of the intervention group (37/72) also received substance abuse counselling

^f^White et al. 2002 [15]: partly blinded

^g^Adjusted OR, adjusted for: treatment condition, recruitment status, binge drinking

^h^Inmates who started treatment in jail and were released before treatment completion

^i^Tulsky et al. 2004 [56]: partly blinded; dissimilarities between treatment arms (primary housing in last year shelter/street; not found to be an independent predictor of completion in this study)this study presents data for incentive vs. another incentive (rather than vs. no incentive)

^j^Adjusted OR, not reported which factors this OR was adjusted for

^k^$25 equivalent in food or transportation vouchers

^l^Total number of events <125

^m^Patients with normal chest X-rays prescribed H, while those with evidence of old TB on chest X-ray were prescribed HR. Participants randomly assigned to the cash or non-cash incentive. Non-cash incentives consisted of a choice of $5 equivalent in fast-food or grocery store coupons, phone cards or bus tokens

**Table 5:** Grading of body of evidence for the effectiveness of social interventions. Question: Do social interventions result in higher initiation, adherence, or completion rates than usual care in individuals eligible for LTBI treatment?

			Quality assessment					n/N = %^a^	Effect		Quality	Importance
No of studies (No of participants)	Design	Population intervention^b^	Risk of bias	Inconsistency	Indirectness	Imprecision	Other considerations	Social intervention	OR (95 % CI)^c^	Absolute^d^ (per 1000 (95 % CI))		
								No social intervention				
Initiation												
1 (946) [18]	Observational study	Immigrants	Not serious^e^	Not serious	Not serious	Not serious	None	389/442 = 88 %	2.7 (1.9–3.8)	149 (107–181)	⊕ ⊕ OO Low	Critical
		Cultural case management						557/762 = 73 %				
Adherence												
								N	*Cumulative mean number of pills taken over 9 months* ^f^			
1 (286) [19]	RCT	General population	Not serious^g^	Not serious	Not serious	Serious^h^	None	92	*180*	–	⊕ ⊕ OO Low	Critical
		Adherence coaching						98	*151*			
1 (184) [57]	Observational study	Immigrants	Not serious^i^	Not serious	Not serious	Serious	None	53	*157*	–	⊕OOO Very low	Critical
		Cultural intervention						131	*129*			
Completion												
3 (928) [19, 27, 58]	RCT	General population	Not serious^j^	Not serious	Not serious	Not serious	None	331/515 = 64 % (range: 46–84 %)	1.4 (1.1–1.9)	78 (53–80)	⊕ ⊕ ⊕O High	Critical
		Counsellor/contingency contracting & adherence coaching/self-esteem counselling & peer based						253/413 = 61 % (range: 38–76 %)				
1 (946) [18]	Observational study	Immigrants	Not serious^e^	Not serious	Not serious	Not serious	None	319/389 = 82 %	7.8 (5.7–10.7)	452 (400–494)	⊕ ⊕ OO Low	Critical
		Case management taking into account cultural background						205/557 = 37 %				
1 (216) [15]	RCT	Inmates^k^	Not serious^l^	Not serious	Not serious	Serious^m^	None	24/106 = 23 %	2.2 (1.0–4.7)^n^	108 (4–267)	⊕ ⊕ O Moderate	Critical
		Education						12/103 = 12 %				
1 (520) [35]	RCT	Homeless	Not serious^o^	Not serious	Not serious	Not serious	None	173/279 = 62 %	3.0 (2.2–4.2)^p^	268 (189–339)	⊕ ⊕ ⊕ ⊕ High	Critical
		Nurse case management						94/241 = 39 %				
1 (199) [17]	RCT	PWID	Not serious^q^	Not serious	Not serious	Not serious	None	79/101 = 78 %	1.0 (0.7–1.5)	2 (−75-62)	⊕ ⊕ ⊕ ⊕ High	Critical
		Peer support vs. no peer support						79/100 = 79 %				

Bibliography: Goldberg et al. 2004 [18]; Hovell et al. 2003 [19]; Ailinger et al. 2010 [57]; Kominski et al. 2007 [27]; Hirsch-Moverman et al. 2013 [58]; White et al. 2002 [15]; Nyamathi et al. 2006 [35]; Chaisson et al. 2001 [17]

n/N: No of individuals with LTBI who initiated, or adhered to or completed treatment/total number of subjects. CI: confidence interval; H: isoniazid; OR: odds ratio; RCT: randomized controlled trial

^a^If >1 articles, weighed pooled point estimates and 95 % CI were calculated

^b^All groups H > 4 months

^c^If >1 articles, pooled estimates and 95%CI were calculated using a random effects model (without quality index)

^d^Calculated via GradePro

^e^Goldberg et al. 2004 [18]: use of unvalidated patient-reported outcomes (self-report); proportion of children aged 5-14 years was higher during one period than the other (19 % vs. 13 %, *p* = 0.003)

^f^No adherence rates were provided as outcome; instead, the cumulative mean number of pills taken per group was presented

^g^Hovell et al. 2003 [19]: unclear allocation concealment; unclear sequence generation; partly blinded. Not downgraded for these risk of bias aspects because already downgraded for imprecision

^h^Total sample size <230

^i^Ailinger et al. 2010 [57]: use of unvalidated patient-reported outcomes (self-report) convenience sample

^j^Hovell et al. 2003 [19]: unclear allocation concealment; unclear sequence generation; partly blinded. Kominski et al. 2007: unclear allocation concealment; no blinding; unclear if intention-to-treat analysis was performed; use of unvalidated patient-reported outcomes (self-report). Hirsch-Moverman et al. 2013: unclear allocation concealment; unclear sequence generation; partly blinded; use of unvalidated patient-reported outcomes (self-report)

^k^Inmates who started treatment in jail and were released before treatment completion

^l^White et al. 2002 [15]: partly blinded

^m^Total number of events <125

^n^Adjusted OR, not reported which factors this OR was adjusted for

^o^Nyamathi et al. 2006 [35]: unclear allocation concealment; unclear sequence generation; partly blinded; dissimilarities between treatment arms (daily alcohol or drug use [significantly associated with non-completion in this study]; male, recruitment site [both not significantly associated with completion in this study], lifetime intravenous drug use, recent self-help program)

^p^Adjusted OR, adjusted for: age, sex, high-school graduate, never married, medical insurance, recruited from homeless shelter, years homeless, treatment completion important, intended to adhere, daily alcohol/drug use, recent self-help program, emotional well-being, social support, recent hospitalization, recent victimization

^q^Chaisson et al. 2001 [17]: unclear allocation concealment; no blinding; use of unvalidated patient-reported outcomes (self-report; urine tests and MEMS in a subset of patients in this study show that self-report is subject to serious under-reporting)

**Table 6:** Grading of body of evidence for effectiveness of other interventions. Question: Do interventions (other than short treatment, incentives or social intervention) result in higher initiation, adherence, or completion rates than usual care in individuals eligible for LTBI treatment?

			Quality assessment					n/N = %	Effect		Quality	Importance
No of studies (No of participants)	Design	Population treatment intervention^a^	Risk of bias	Inconsistency	Indirectness	Imprecision	Other considerations	Other intervention	OR (95 % CI)	Absolute^a^ (per 1000 (95 % CI))		
								Usual care				
Initiation												
1 (107) [59]	Observational study	Healthcare workers H	Not serious^b^	Not serious	Not serious	Serious^c^		32/62 = 52 %	8.8 (3.1–23)	413 (168–631)	⊕OOO Very low	Critical
		Use of IGRAs						5/45 = 11 %				
Adherence												
0 (0)	No evidence available	–	–	–	–	–	–	–	*–*	–	–	Critical
Completion												
0 (0)	No evidence available	–	–	–	–	–	–	–	–	–	–	Critical

Bibliography: Sahni et al. 2009 [59]

*n/N* No of individuals with LTBI who initiated, or adhered to or completed treatment/total number of subjects; *CI* confidence interval; *IGRAs* Interferon Gamma Release Assay; *OR* odds ratio; *PWID* people who inject drugs; *RCT* randomised controlled trial

^a^Calculated via GradePro

^b^Use of unvalidated patient-reported outcomes (telephone interview)

^c^Total number of events <125

#### Does short LTBI treatment result in higher initiation, adherence, or completion rates than long LTBI treatment in individuals eligible for LTBI treatment (Table 2)?

**Fig. 1:**
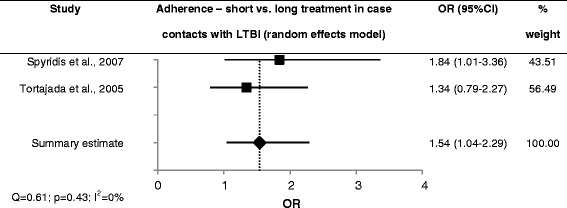
Forest plot for adherence to short vs. long LTBI treatment in case contacts with LTBI

**Fig. 2:**
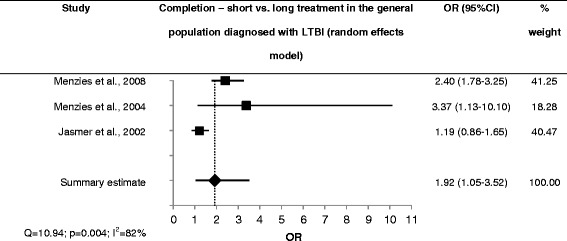
Forest plot for completion of short vs. long LTBI treatment in the general population diagnosed with LTBI

Case contacts showed better adherence when receiving short treatment (2 studies; sOR = 1.5; 95 % CI 1.0-2.3; low heterogeneity, moderate quality of evidence) [21, 50] (Fig. 1). One of these studies also provided completion rates but found no association with shorter treatment duration (OR = 0.8; 95 % CI 0.5–1.3; moderate quality of evidence) [21]. All other studies found higher completion rates in the short treatment group: in immigrants with LTBI (1 study; OR = 2.5; 95 % CI 1.7–3.6; moderate quality of evidence) [38], in the general population with LTBI (2 studies; sOR = 1.9; 95 % CI 1.1–3.5; large heterogeneity, moderate quality of evidence) [51–53] (Fig. 2), and in case contacts (1 study; OR = 2.1; 95 % CI 1.9–2.3; low quality of evidence) [20]. The latter result is confounded, however, by the use of DOT in the short treatment group and SAT in the long treatment group.

#### Does DOT result in higher initiation, adherence, or completion rates than SAT in individuals eligible for LTBI treatment (Table 3)?

In undocumented migrants, significantly lower completion rates were found among those receiving twice weekly clinic-based DOT compared to daily SAT (OR = 0.1; 95 % CI 0.0–0.3; low quality of evidence) [54] or twice weekly SAT (OR = 0.2; 95 % CI 0.1–0.6). In one other study, in PWID, no effect of DOT administered by an outreach nurse on completion rates of LTBI treatment was found (OR = 1.1; 95 % CI 0.5–2.1; moderate quality of evidence). However, when looking at the proportion of people who took all doses, the DOT group performed significantly better (OR = 31.5; 95 % CI 14.1–70.6) [17].

Two more studies compared DOT to SAT, but were confounded: among case contacts with LTBI a shorter treatment regimen was given in the DOT than in the SAT group [20], and among PWID with LTBI the DOT group received methadone treatment whereas the SAT group did not [16]. Higher completion rates were found in the DOT group among both case contacts (OR = 2.1; 95 % CI 1.9–2.3; low quality of evidence) and PWID (OR = 14.5; 95 % CI 5.1–42; very low quality of evidence).

#### Does treatment supported by (monetary) incentives result in higher initiation, adherence, or completion rates than treatment not supported by incentives in individuals eligible for LTBI treatment (Table 4)?

Two studies in PWID with LTBI found higher completion rates for LTBI treatment among those who received either a monetary incentive (adjusted OR [aOR] = 32.0; 95 % CI 7.1–145; moderate quality of evidence) [55] or methadone treatment (OR = 14.5; 95 % CI 5.0–42; very low quality of evidence) [16] compared to those who received no incentive. The results from the methadone treatment study were confounded, however, by the use of DOT in the methadone treatment group and SAT in the control group. The provision of food or transportation vouchers to released inmates with LTBI if they attended a TB clinic upon release (OR = 1.1; 95 % CI 0.5–2.4; moderate quality of evidence) [15] did not lead to better completion rates. In another study, no difference was found between the provision of cash-incentives versus non-cash-incentives to homeless individuals with LTBI (OR = 1.7; 95 % CI 0.7–4.3; low quality of evidence) [56].

#### Do social interventions result in higher initiation, adherence, or completion rates than standard care in individuals eligible for LTBI treatment (Table 5)?

**Fig. 3:**
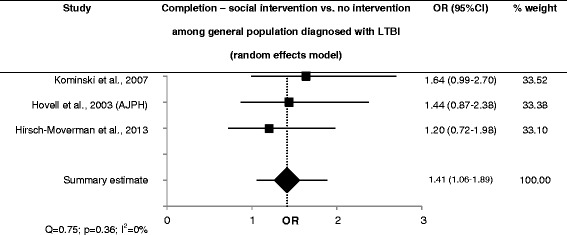
Forest plot for completion of LTBI treatment using social interventions in the general population diagnosed with LTBI

Adherence coaching among the general population with LTBI at clinics (low quality of evidence) and a cultural intervention among immigrants (very low quality of evidence) resulted in better adherence [19, 57]. Social interventions were found to improve completion rates of LTBI treatment compared to the standard care group in all but one study, which provided peer-support among PWID with LTBI (OR = 1.0; 95 % CI 0.7–1.5; high quality of evidence) and found no effect on completion [17]. Counselling and contingency contracting, adherence coaching and self-esteem counselling, and peer-based interventions in the general population showed better completion rates (sOR = 1.4; 95 % CI 1.1–19; low heterogeneity, high quality of evidence) [19, 27, 58] (Fig. 3).

Education among inmates (OR = 2.2; 95 % CI 1.0–4.7; moderate quality of evidence) [15], nurse case management among homeless individuals (aOR = 3.0; 95 % CI 2.2–4.2; high quality of evidence) [35], and case management with attention for the cultural background of each individual among immigrants (aOR = 7.8; 95 % CI 5.7–10.7; low quality of evidence) [18] improved completion. The latter study also found that this intervention led to higher initiation rates (OR = 2.7; 95 % CI 1.9–3.8; low quality of evidence).

#### Do interventions other than short treatment, directly observed therapy, incentives or social interventions result in higher initiation, adherence, or completion rates than standard care in individuals eligible for LTBI treatment (Table 6)?

One study showed that the use of IGRAs rather than TSTs for diagnosis of LTBI improved the initiation rate of LTBI treatment in healthcare workers with LTBI (OR = 8.8; 95 % CI 3.1–23; very low quality of evidence) [59].

Three retrospective studies performed in the general population with LTBI showed significantly higher completion rates in the groups that received DOT, behaviour modification techniques in combination with incentives, or home to clinic follow-up, respectively [28, 44, 60] (see Additional file 4).

## Discussion

To the best of our knowledge, this is the first review to systematically and comprehensively summarise data on any determinant of, and intervention to improve, LTBI treatment initiation, adherence and completion in all types of populations.

### Determinants

The most frequently found determinants of treatment completion in our review were patient-related (i.e. type of population with LTBI, demographic factors, drug/alcohol abuse), therapy-related (e.g. short therapy regimens, DOT, occurrence of adverse events), and socio-economic (e.g. unemployment, lack of social support). Unfavourable socio-economic factors were consistently associated with poor completion of LTBI therapy. These results should be interpreted with care, since different measures of associations were used in the studies, the reference groups varied between studies, and data on non-significant factors were not always quantified in the studies and were therefore not listed in this review. However, the same socio-economic factors were also predictors of non-adherence to highly active antiretroviral therapy in human immunodeficiency virus (HIV)-patients or to cardiovascular medication [61–65]. Also, adverse events have been associated with worse adherence to treatment for all these conditions [61, 62]. Similar factors were associated with adherence to treatment for active TB [61].

### Interventions

#### Initiation

Some evidence was found that the use of IGRAs rather than TSTs [59], or a social intervention using case management with attention to an individual’s cultural background might positively influence the initiation rate of LTBI treatment [18].

#### Adherence

Our meta-analysis showed that case contacts had better adherence if they received short treatment compared to those on long treatment regimens [21, 50]. Social interventions in the form of adherence coaching of adolescents with LTBI and cultural interventions among immigrants with LTBI also resulted in improved adherence [19, 57].

#### Completion

Overall, completion rates of LTBI treatment were better among groups receiving shorter regimens than those with longer treatment regimens. The only outcome regarding the effect of shorter treatment on completion rates for which no effect was found could be explained by a relatively high rate of hepatotoxicity (11 %) found in the short treatment arm compared to the long treatment arm (3 %) [21]. This led to premature termination of the study. The applied short regimen of rifampicin plus pyrazinamide is currently not generally offered to persons with LTBI due to its association with hepatotoxicity [66].

Mixed results were found on the effect of DOT on completion rates of LTBI treatment. The significantly lower completion rates among those receiving clinic-based DOT in one study might be attributable to the difficulty undocumented migrants have in reporting regularly to health services to collect their drugs [54]. The study in which no effect was found among PWID did find that more people in the DOT group took all doses and, importantly, also found that the number of self-reported doses taken was likely to be greatly over-estimated [17]. With regards to two other types of long-term treatment, highly active antiretroviral therapy in HIV-patients and TB treatment, no overall benefit of DOT compared to SAT was found on viral load among HIV-patients or cure among TB patients in reviews by Nachega et al. and Volmink et al. [67, 68].

Of the studies reporting on the effect of incentives, two studies conducted in PWID with LTBI found a positive result (one of which was confounded), and the other study in released inmates found no effect. The success of incentives is likely to be both population and setting dependent. Lutge et al. reviewed the literature on material incentives and treatment for latent TB or active TB disease and concluded that the effect on long-term adherence and completion is not clear [69].

Social interventions to improve LTBI treatment uptake included case management with attention for an individual’s cultural background, adherence coaching, counselling, contingency contracting, education, nurse case management and peer-based interventions. Most studies on this topic showed better completion rates in the intervention group than in the standard-care group, regardless of the type of social intervention. In a review of RCTs, Schroeder et al. found patient education to be largely unsuccessful in improving adherence to blood pressure-lowering medication, whereas some motivational strategies and complex interventions were successful [70]. In three out of the seven RCTs in a review of Schedlbauer et al. social interventions (i.e. patient information and education, intensified patient care, or a complex behavioral approach) improved adherence rates to lipid-lowering medication) [71].

### Other reviews

Several other reviews present data on interventions to improve medication uptake among LTBI patients. These reviews had specific questions, for example the effect of lay healthcare workers on completion of LTBI treatment [72], interventions to improve the health of the homeless [73], education or counselling to improve completion of LTBI treatment [7], effects of rifampicin monotherapy or rifamycin-combination therapy versus isoniazid for preventing active TB and the role of completion rates [9]; incentives to reinforce medication adherence, including for LTBI treatment [10, 69]. However, these reviews included only a small number of studies with the specific aim to investigate interventions to improve LTBI treatment initiation, adherence or completion. Some of these articles were also included in the current review; some could not be included in our review because we used different inclusion criteria.

### Limitations

The definitions of completion varied between the included studies, as did the ways in which treatment adherence was assessed and in which completion rates were calculated. For example definitions used varied from “completed four months of rifampicin” to “picked up nine months of isoniazid within twelve months” and “took at least 80 % of the prescribed medication within twenty weeks”. This heterogeneity complicates comparison of rates between studies and hampers meta-analysis and interpretation of the results. Since adherence and completion are similar concepts in the sense that full adherence to a treatment regimen leads to its completion, the limitations that are applicable to measures for completion are also applicable to adherence.

There is no standard definition for LTBI using tuberculin reactivity which is universally accepted.

There were no pre-set LTBI diagnostic criteria for inclusion of the studies in this review, the inclusion relied on reporting of the diagnostic criteria of the individual studies; if the study considered a case to be diagnosed then the study was included in the review and those cases were analysed.

Only determinants that showed a statistically significant association with initiation, adherence and completion were listed in this article, this should be taken into account when interpreting the results. The power of a study to detect a significant effect was not taken into account in this review. Additionally, the determinants were merely described and no summary analyses were done because the non-significant determinants were not quantified and because of heterogeneity between the included studies. Comparison of studies is also complicated by the fact that different measures of association were used by the included studies; reference groups may differ; or the definition of the determinant itself might vary. These intricacies would be lost when grouping the determinants.

We calculated summary estimates for studies with similar populations and interventions. However, the studies were still quite heterogeneous. Calculation of summary estimates by combining studies without correcting for possible bias-causing factors between studies may cause bias in the results. Furthermore, the I^2^ estimates need to be interpreted with caution because each meta-analysis only included three studies.

Finally, when conducting a systematic review, any limitations of included studies (e.g. lack of controlling for relevant covariates) inherently become limitations of data presented in the review.

### Gaps and future research

The number of intervention studies in specific populations was scarce. In order to generate evidence on the effectiveness of context-specific interventions to improve the uptake of LTBI treatment, RCTs tailored to specific populations, with consideration of available resources and infrastructure of the health system, are necessary [11]. Although clinically relevant, no determinants of initiation, adherence or completion were found for patients with comorbidities.

Ultimately, the effect of LTBI treatment on the development of TB disease is important, and initiation, adherence and completion rates of LTBI treatment are intermediate determinants. In a subgroup analysis of a network meta-analysis of RCTs to determine the most efficacious regimen for preventing active TB disease [74], no evidence of a relationship between adherence (expressed as overall percentage of doses received) and efficacy was found. Still, more information on the association between treatment adherence and efficacy for prevention of active TB may be valuable.

## Conclusions

Clinical benefit to individuals with LTBI and the success of the LTBI control programme in general are dependent on individuals taking the medication and completing the full course of treatment [11]. In the first part of this review, it was found that initiation and completion rates of LTBI treatment were frequently suboptimal and varied greatly within and across different populations. Taking determinants of initiation, adherence, and completion into account is an important first to step to plan interventions to improve these rates.

The available evidence on the effect of interventions on treatment initiation, adherence and completion presented in this review suggests that some interventions, notably the use of shorter treatment regimens and social interventions, have a positive effect on adherence and completion.

Overall, however, the evidence was inconclusive and recommendations on the best interventions to improve uptake of LTBI medication are hampered by the heterogeneity of the studies. The benefit of interventions to improve treatment completion, such as incentives and DOT, appears to be population and setting dependent. Specific needs of the different populations with LTBI should be addressed taking into consideration the local context, specific settings and conditions in which the LTBI treatment programme is implemented.

## Ethics approval and consent to participate

Not applicable.

## Consent for publication

Not applicable.

## Availability of data and materials

The datasets supporting the conclusions of this article are included within the article and its additional files.

## Additional files

Additional file 1: Materials and methods. (DOCX 47 kb) Additional file 2: Flow chart of selection process. (DOCX 257 kb) Additional file 3: Study characteristics, outcomes, and quality aspects of risk of bias assessment of articles on determinants of initiation, adherence and completion of LTBI treatment regimens. (DOCX 151 kb) Additional file 4: Study characteristics, outcomes, and risk of bias assessment of articles on interventions to improve initiation, adherence and completion of LTBI treatment regimens. (DOCX 104 kb)

## Abbreviations

DOT: directly observed therapy
EU/EEA: European Union and European Economic Area
GRADE: Grading of Recommendations Assessment, Development and Evaluation
HIV: human immunodeficiency virus
IGRAs: interferon gamma release assay
LTBI: latent tuberculosis infection
ORs: odds ratios
PWID: people who injected drugs
RCT: randomized controlled trial
SAT: self-administered therapy
TB: tuberculosis
TST: tuberculin skin tests
WHO: World Health Organization
