# Supplementary material for: Interventions for improving adherence to treatment for latent tuberculosis infection: a systematic review
Source: BMC Infect Dis. 2016 Jun 8;16:257. doi: 10.1186/s12879-016-1549-4 (PMC4897858; doi:10.1186/s12879-016-1549-4)
Supplement: Additional file 1: — Materials and methods. (DOCX 47 kb) [file 12879_2016_1549_MOESM1_ESM.docx]

## Additional file 1 – Materials and methods

A systematic literature review was performed according to a review protocol and following the Cochrane guidelines. The aim of the systematic review was to provide answers to the following research questions: 1) What is the LTBI treatment initiation rate and the completion rate for each recommended LTBI treatment regimen; 2) What are determinants of LTBI treatment initiation, adherence, and completion; 3) What are the interventions with demonstrated efficacy or effectiveness to improve LTBI treatment initiation, adherence and completion in individuals who are eligible for LTBI treatment. The results for review questions 2 and 3 are presented in this article. PICO (Population-Intervention-Comparator-Outcome) questions were formulated based on the review questions.

The following PICO questions were used to define the search string:

| **2a** | **What are the determinants of LTBI treatment initiation?** |
| --- | --- |
| P | Individuals with indication for LTBI treatment |
| I | Individuals who initiate LTBI treatment |
| C | Individuals who do not initiate LTBI treatment |
| O | Determinants of treatment initiation |

| **2b** | **What are the determinants of LTBI treatment adherence?** |
| --- | --- |
| P | Individuals who initiate LTBI treatment |
| I | Individuals who adhere to LTBI treatment |
| C | Individuals who do not adhere to LTBI treatment |
| O | Determinants of treatment adherence |

| **2c** | **What are the determinants of LTBI treatment completion?** |
| --- | --- |
| P | Individuals who initiate LTBI treatment |
| I | Individuals who complete LTBI treatment |
| C | Individuals who do not complete LTBI treatment |
| O | Determinants of treatment completion |

| **3** | **In individuals who are eligible for LTBI treatment, what are the interventions with demonstrated efficacy or effectiveness to improve LTBI treatment initiation, adherence and completion?** |
| --- | --- |
| P | Individuals with indication for LTBI treatment |
| I | Individuals with LTBI treatment receiving the intervention to improve LTBI treatment initiation, adherence and/or completion |
| C | Individuals with LTBI treatment not receiving the intervention to improve LTBI treatment initiation, adherence and/or completion |
| O | Proportion of eligible individuals who initiated, adhered and/or completed treatment  Other outcomes for efficacy or effectiveness, acceptability and feasibility of the intervention |

***Eligibility criteria***

Only primary articles describing randomised controlled trials (RCTs), non-randomised prospective comparative studies of interventions, prospective longitudinal observational studies, and retrospective studies were included in this review. Systematic reviews were not included; however the reference lists of relevant systematic reviews were screened to find primary articles that were not found via our literature search. Studies in individuals eligible for LTBI treatment were considered relevant. Eligibility for LTBI treatment was defined as “being diagnosed with LTBI”. There was no required minimum study duration or number of subjects, except for studies in the general population diagnosed with LTBI that also presented data stratified for specific populations (e.g. case contacts, immigrants etc.). Since these studies were primarily aimed at the total population, and sampling strategies were applied accordingly, data for the specific populations were only extracted when such a population consisted of at least 30 subjects. For an article to be included in the review, baseline data (e.g. population characteristics) must be presented, LTBI had to be defined in the study (e.g. as “positive tuberculin skin tests (greater of equal 10 mm) and negative chest radiographs”) and the LTBI treatment regimen had to be specified, and for studies presenting completion rates, a definition for “completion” had to be provided. Adherence rates that met the definition of “completion” (e.g. “full adherence” or “adherence for nine months”) were interpreted as completion rates. If individuals whose completion status was pending by the end of a study were included in the completion rate, the rate was recalculated to exclude these individuals. Studies that included only case contacts who received chemoprophylaxis irrespective of whether or not LTBI was diagnosed were excluded from this review.

***Information sources and search strategy***

We searched the databases PubMed and Embase. Search strings were composed for 1) LTBI, 2) LTBI treatment, and 3) initiation, adherence, completion and implementation. A fourth search string was composed to exclude animal studies. No geographical, time, or language limits were applied, however only full-text articles in English, French, Spanish, German, and Dutch were reviewed. The search was carried out on February 3^rd^, 2014 for all literature published up to that date. Output from the searched databases was exported to Endnote version X4.0.2.

***Search string*PubMed**

*#A Search string for LTBI:*

“latent tuberculosis” [Mesh] OR “latent tuberculosis” [tiab] OR LTB [tiab] OR LTBI [tiab] OR ((laten* [tiab] OR dorman* [tiab]) AND (TB [tiab] OR tuberc* [tiab]))

*#B Search string for LTBI treatment:*

"therapeutics" [Mesh] OR "therapy" [Subheading] OR "treatment outcome" [Mesh] OR "primary prevention" [Mesh] OR "secondary prevention" [Mesh] OR “prevention and control” [Subheading] OR treatment* [tiab] OR therapy [tiab] OR therapies [tiab] OR therapeutics [tiab] OR prevent* [tiab] OR management [tiab] OR “antibiotic prophylaxis” [Mesh] OR “chemoprevention“ [Mesh] OR prophyla* [tiab] OR chemoprophylaxis [tiab] OR DOT [tiab] OR DOTS [tiab] OR "isoniazid" [Mesh] OR isoniazid [tiab] OR INH [tiab] OR IPT [tiab] OR "rifapentine" [Supplementary Concept] OR rifapentine [tiab] OR RPT [tiab] OR "rifampin" [Mesh] OR rifampin [tiab] OR RIF [tiab] OR rifampicin [tiab] OR ethambutol [tiab] OR EMB [tiab] OR ethionamide [tiab] OR ETH [tiab] OR pyrazinamide [tiab] OR PZA [tiab] OR fluroquinolones [tiab] OR FLQ [tiab] OR moxifloxacin [tiab] OR levofloxacin [tiab] OR gatifloxacin [tiab]

*#C Search string for adherence, initiation, completion, and implementation:*

"attitude" [Mesh] OR adher* [tiab] OR “medication adherence” [Mesh] OR “guideline adherence” [mesh] OR “patient compliance” [mesh] OR complian* [tiab] OR comply* [tiab] OR accordance [tiab] OR according [tiab] OR agreement [tiab] OR “withholding treatment” [mesh] OR initiat* [tiab] OR start [tiab] OR commenc* [tiab] OR begin* [tiab] OR introduc* [tiab] OR enroll* [tiab] OR complet* [tiab] OR finaliz* [tiab] OR finalis* [tiab] OR fulfill* [tiab] OR ending [tiab] OR finish* [tiab] OR terminat* [tiab] OR accomplish* [tiab] OR realiz* [tiab] OR realis* [tiab] OR attain* [tiab] OR implement* [tiab] OR apply* [tiab] OR application* [tiab] OR “medication therapy management” [mesh]

*#D Search string for animal studies:*

Animals [Mesh] NOT (Humans [Mesh] AND Animals [Mesh])

*Search:*

((#A AND #B AND #C) NOT #D)

**EMBASE**

*#A Search string for LTBI:*

“latent tuberculosis”/exp OR (tuberculosis/exp AND 'latent period'/de) OR (LTB OR LTBI OR ((laten* OR dorman*)  AND (TB OR tuberc*))):ab,ti

*#B Search string for LTBI treatment:*

“therapy”/exp OR therapy:lnk OR “treatment outcome”/exp OR “primary prevention”/exp OR "prevention and control"/de OR “secondary prevention”/exp OR prevention:lnk OR “antibiotic prophylaxis”/exp OR “chemoprophylaxis”/exp OR “isoniazid”/exp OR “rifampicin”/exp OR (treatment* OR therap* OR prevent* OR management OR control OR prophyla* OR chemoprophyla* OR DOT OR DOTS OR isoniazid OR INH OR IPT OR rifapentine OR RPT OR rifampin OR RIF OR ethambutol OR EMB OR ethionamide OR ETH OR pyrazinamide OR PZA OR fluroquinolones OR FLQ OR moxifloxacin OR levofloxacin OR gatifloxacin):ab,ti

*#C search string for adherence, initiation, completion and implementation:*

“medication compliance”/exp OR “patient attitude”/de OR “treatment withdrawal”/exp OR “medication therapy management”/exp OR "treatment refusal"/exp OR "refusal to participate"/de OR (adher* OR complian* OR comply* OR accordance OR according OR agreement OR Initiat* OR start* OR commenc* OR begin OR introduc* OR enroll* OR complet* OR finaliz* OR finalis* OR fulfill* OR ending OR finish* OR terminat* OR accomplish* OR realiz* OR realis* OR attain* OR implement* OR apply* OR application* OR refus* OR attitud*):ab,ti

*#D Search string for animal studies:*

(“animal”/exp NOT “human”/exp)

*Search:*

((#A AND #B AND #C) NOT #D)

***Study selection***

Articles were selected by a three-step selection procedure based on 1) screening of title and abstract, 2) screening of full-text article, and 3) final screening during the data-extraction phase. One-hundred percent of the title and abstract selection and critical appraisal of the full-text articles was done in duplicate by two independent researchers; the results were compared and discussed and any doubts were resolved by a third researcher.

***Risk of bias assessment***

The risk of bias of each included full-text article was assessed with standardised, study-design specific, quality appraisal forms following the risk of bias assessment proposed by the Cochrane Collaboration^[[1]](#footnote-1)^. A few additional aspects, not mentioned in the Cochrane Collaboration, were considered when evaluating the quality of the articles, i.e. the adequacy of recall assessment and reporting, whether confidence intervals were provided, and for retrospective studies, the adequacy of the method of retrospective selection of the population. For review questions 2 and 3 it was also determined whether confounders were taken into account. Each aspect was evaluated as high risk of bias, moderate or unclear risk of bias, or low risk of bias. Because of the descriptive nature of the review question, risk of bias was only assessed for aspects of the individual studies, without providing an overall level of quality for each individual study. As review question 1 does not deal with the effects of health interventions and treatment and populations vary widely between studies, risk of bias was also not assessed across the evidence base per outcome.

***Data extraction***

Evidence tables were compiled by two researchers and reviewed by a third researcher. The data extraction was done in duplicate for 15% of the included articles, no major differences were found. Evidence tables were created for different populations with LTBI: 1) general population (primarily unselected individuals with LTBI at clinics), 2) case contacts, 3) healthcare workers, 4) the homeless, 5) people who inject drugs (PWID), 6) HIV-infected individuals, 7) inmates, 8) immigrants, and 9) patients with comorbidities, e.g. patients with rheumatoid arthritis or patients with hematologic malignancies. The definitions of completion were those used in the individual studies; there were differences in definitions between studies. Study results were sorted by study design and split by duration of the LTBI treatment regimen, i.e. short (≤four months), long (>four months), or short and long combined when no data were presented for short and long LTBI treatment separately.

***Synthesis of results***

For data visualisation, forest plots of initiation and completion rates were created for the identified populations in Excel 2010^[[2]](#footnote-2)^. The MetaXL 2.1 add-in in Excel was used to calculate 95% confidence intervals around initiation and completion rates. We planned to calculate pooled rates, however this was not done for the first review question, because of the large heterogeneity of the included articles.

1. Higgins JP, Altman DG, Gotzsche PC, et al. The Cochrane Collaboration's tool for assessing risk of bias in randomised trials. BMJ 2011; 343:d5928. [↑](#footnote-ref-1)
2. Neyeloff JL, Fuchs SC, Moreira LB. Meta-analyses and Forest plots using a microsoft excel spreadsheet: step-by-step guide focusing on descriptive data analysis. BMC research notes 2012; 5:52. [↑](#footnote-ref-2)
