# Supplementary material for: Interventions for improving adherence to treatment for latent tuberculosis infection: a systematic review
Source: BMC Infect Dis. 2016 Jun 8;16:257. doi: 10.1186/s12879-016-1549-4 (PMC4897858; doi:10.1186/s12879-016-1549-4)
Supplement: Additional file 2: — Flow chart of selection process. (DOCX 257 kb) [file 12879_2016_1549_MOESM2_ESM.docx]

##
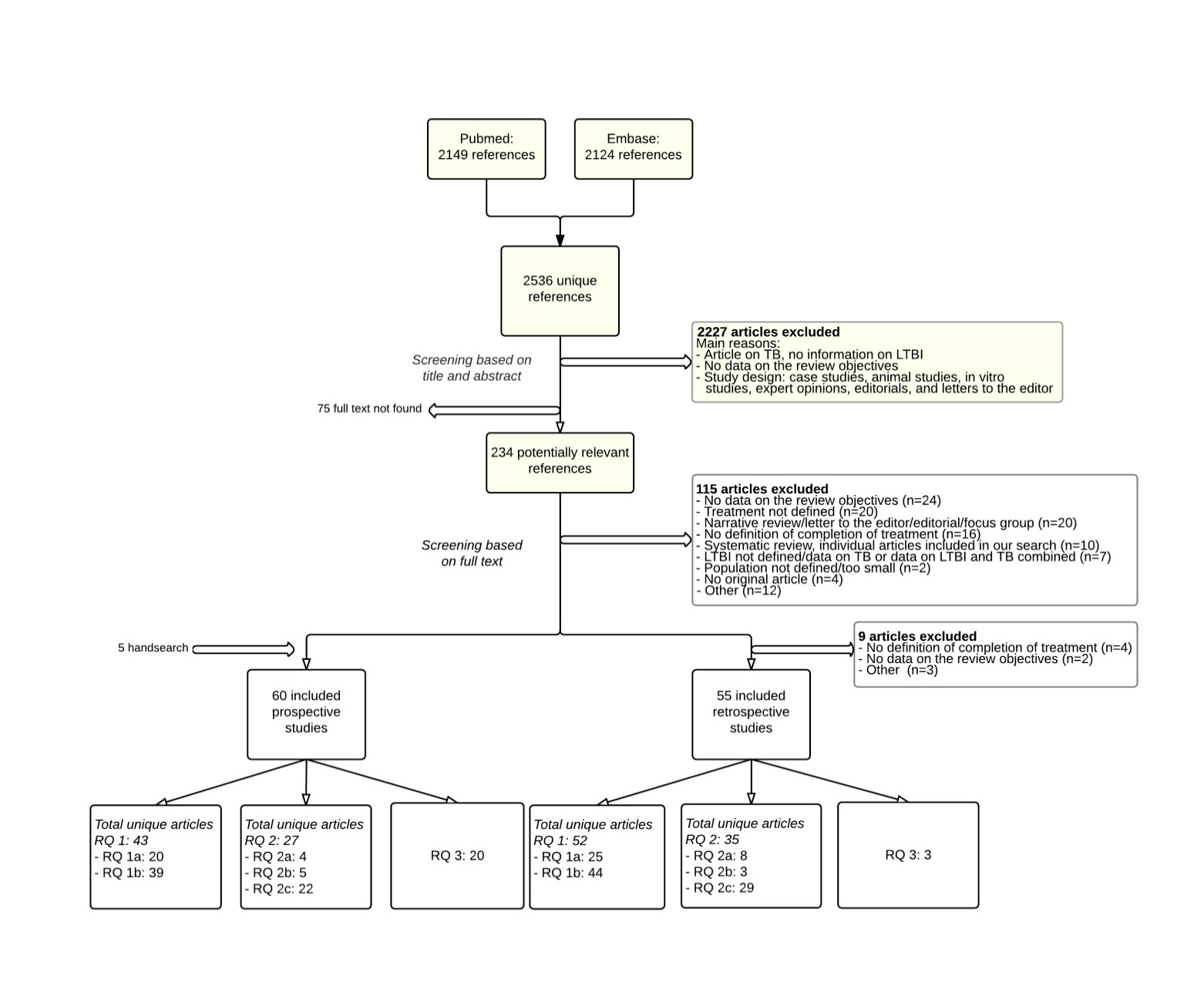
Additional file 2– Flow chart of selection process

RQ: review question. LTBI: latent tuberculosis infection; TB: tuberculosis. Review question 1a: What is the initiation rate for each recommended LTBI treatment regimen?; Review question 1b: What is the completion rate for each recommended LTBI treatment regimen?; Review question 2a: What are the determinants of LTBI treatment initiation?; Review question 2b: What are the determinants of LTBI treatment adherence?; Review question 2c: What are the determinants of LTBI treatment completion?; Review question 3: In individuals who are eligible for LTBI treatment, what are the interventions with demonstrated efficacy or effectiveness to improve LTBI treatment initiation, adherence and completion?
