# Supplementary material for: Interventions for improving adherence to treatment for latent tuberculosis infection: a systematic review
Source: BMC Infect Dis. 2016 Jun 8;16:257. doi: 10.1186/s12879-016-1549-4 (PMC4897858; doi:10.1186/s12879-016-1549-4)
Supplement: Additional file 3: — Study characteristics, outcomes, and quality aspects of risk of bias assessment of articles on determinants of initiation, adherence and completion of LTBI treatment regimens. (DOCX 151 kb) [file 12879_2016_1549_MOESM3_ESM.docx]

## Additional file 3– Study characteristics, outcomes, and quality aspects of risk of bias assessment of articles on determinants of initiation, adherence and completion of LTBI treatment regimens

| **Reference, country** | | **Study design** | | **Study population**  **(sample size)** | | **Determinants** | | | **Quality aspects risk of bias assessment of each study** |
| --- | --- | --- | --- | --- | --- | --- | --- | --- | --- |
|  |  |  |  |  |  | **Initiation**  **Treatment, measure of association, results** | **Adherence**  **Treatment regimen (months-type), measure of association, results** | **Completion**  **Treatment regimen (months-type), measure of association, results** |  |
| ***Randomised controlled trials*** | | | | | | | | | |
| Bastos et al. 2013 [1]  Brazil | | International multicenter clinical trial (parent trial) | | General population (with at least one risk factor for progression to disease) (n=160) | | - |  | 4R/9H, SAT  *Completion*  Among individuals that underwent three QFT-GIT (n=68):   - Higher (vs. lower) age: p=0.048   Among individuals who underwent at least one serial QFT-GIT (n=92):   - Higher (vs. lower) age: p=0.04 - Indication for LTBI treatment was immunosuppression (vs. case contact)): p=0.004 - Smoking (vs. non-smoking): p=0.02   R (vs. H): p<0.001 | Low: 6, 8  Moderate: 1, 2, 3, 4, 7  High: 5 |
| Batki et al. 2002 [2]  USA | | Controlled trial | | PWID  (n=111) | | - | 6H, DOT/SAT  *Non-adherence*   - Diagnosis of current major depression (vs. no such diagnosis): p=0.026 | - | Low: 1, 2, 5, 6  Moderate: -  High: 3, 4, 7, 8^J^ |
| Hirsch-Moverman et al. 2013 [3]  USA | | RCT | | General population (n=250) | | - | - | 9H, SAT  Multivariate RR (95% CI)  *Completion*   - Age ≥ 40 years (vs. <40): 1.30 (1.05-1.61); p=0.015 | Low: 1, 4, 5, 6, 7, 8  Moderate: 2, 3  High: - |
| Hovell et al. 2003 [4]  USA | | RCT | | General population (n=286) | | - | 9H, SAT  Multiple regression  *Adherence*   - Higher (vs. lower) grades in school: p=0.0045 - Bicultural (vs. Hispanic or non-Hispanic): p=0.02   *Non-adherence*   - Higher (vs. lower) age: p<0.0001 - Risk behaviours (summing reports of having ever tried alcohol, cigarettes, marijuana, been expelled or suspended from school, or been in a physical fight): p=0.02 | - | Low: 5, 7, 8  Moderate: 1, 2, 3, 4, 6  High: - |
| Jasmer et al. 2002 [5]  USA | | Multicenter, prospective open-label clinical trial | | General population (n=589) | | - | - | 2RZ/6H, SAT  Multivariable: OR (95% CI)  *Non-completion*   - Study site 2 (ref n.r.): 2.06 (1.32- 3.23); p=0.002 - Study site 3 (ref n.r.): 3.48 (2.19-5.53); p<0.001 - Grade 3 or 4 hepatotoxicity (ref n.r.): 4.85 (1.61-14.7) p=0.005 - Adverse events other than hepatotoxicity (ref n.r.): 2.90 (1.83-4.58); p=0.005   *Completion*   - Grade 1 or 2 hepatotoxicity (ref n.r.): 2.81 (1.45-5.45); p=0.002 | Low: 5, 8  Moderate: 2, 3, 6  High: 1, 4, 7 |
| Jiménez-Fuentez et al. 2013 [6]  Spain | | RCT | | Immigrants  (n=590) | | - | - | 3HR/6H, SAT  Univariate: OR (95% CI)  *Incompletion*   - Recruited from screening (vs. recruited via case contacts): 1.88 (1.26-2.82); p=0.001 - Unemployment (vs. employment): 1.91 (1.28-2.85); p=0.0008 - Undocumented migrants (vs. documented migrants): 1.48 (1.01-2.15); p=0.03 - Low education level (vs. middle/ upper level: 1.73 (1.04-2.88); p=0.02 - Not living with family (vs. living with family): 3.7 (2.54-5.4); p<0.001 | Low: 1, 6, 7  Moderate: 2, 3  High: 4, 5, 8^A^ |
| Kominski et al. 2007 [7]  USA | | RCT | | General population (n=794) | | - | - | ≥6H, SAT  Multivariate: OR (95% CI)  *Completion*   - Home situation (vs. living with both natural parents)   Child living with one natural parent:  0.45 (0.29-0.71)  Child living with no natural parent:  0.37 (0.19-0.57)   - Born in USA (vs. foreign born): 0.52 (0.33-0.81) | Low: 1, 4, 8  Moderate: 2, 3, 5, 6  High: 7 |
| Malotte et al. 2001 [8]  USA | | RCT | | PWID  (n=163) | | - | - | 6H/12H, DOT  Multivariate: OR (95% CI)  *Completion*   - Prior study participant (vs. newly recruited): 2.5 (1.1-5.7) | Low: 2, 4, 5, 7, 8  Moderate: 1, 3, 6  High: - |
| Nyamathi et al. 2006 [9]  USA | | Two-group site-randomised design | | Homeless individuals  (n=520) | | - | - | 6H, DOT  Multivariable: OR (95% CI)  *Completion*   - Age (by one unit increment): 1.03 (1.01-1.06) - High-school graduate (vs. persons with <12y of formal education): 1.71 (1.22-2.39) - Never married (vs. married, divorced, separated or widowed): 1.89 (1.56-2.28) - Medi-Cal insurance (vs. Medicare, health maintenance organization, veterans, private other or no insurance): 2.05 (1.15-3.65) - Years homeless; log (by one unit increment): 1.28 (1.05-1.57) - Strongly agree treatment completion important (vs. mildly agree, mildly disagree or strongly disagree): 3.08 (1.28-7.42) - Intended to adhere (vs. probably would adhere, may adhere, probably would not or definitely would not adhere): 1.87 (1.30-2.67) - Daily alcohol/ drug use (vs. less frequent or no use): 0.45 (0.33-0.62) - Recent self-help program (vs. no recent attendance at informal substance abuse treatment meetings): 1.39 (1.12-1.72) - Social support (by one unit increment): 1.01 (1.00-1.01) - Recent hospitalization (vs. no recent hospitalization): 0.50 (0.27- 0.95) - Recent victimization (vs. no physical or sexual assault in past 6 months): 2.19 (1.40-3.44) | Low: 1, 5, 6, 7  Moderate: 2  High: 3, 4, 8 |
| Trajman et al. 2010 [10]  Canada, Saudi Arabia  (same study as Menzies et al. 2008) | | RCT | | General population (n=802) | | - | - | 4R/9H, SAT  Multivariate: OR (95% CI)  *Completion*   - Recruitment centre 9 (vs. recruitment centre 1): 0.2 (0.1-0.4) | Low: 1, 4, 6, 7, 8  Moderate: 2  High: 3, 5 |
| Tulsky et al. 2004 [11]  USA | | RCT | | Homeless individuals  (n=119) | | - | - | 4HR/6H, DOT/SAT  Multivariate: OR (95% CI)  *Completion*   - Male: 5.65 (1.36-23.40); p=0.02 - Residence in hotel/other at study entry (vs. shelter/street): 4.86 (1.32- 17.94); p=0.02 | Low: 1, 2, 6  Moderate: 3, 4  High: 5, 7, 8 |
| ***Prospective observational studies*** | | | | | | | | | |
| Berg et al. 2004 [12]  USA | Prospective cohort study | | General population (n=96) | | | - | 9H, SAT  Multiple regression  *Non-adherence*   - Sum somatic complaints over 9 mo.: p=0.037 - Sum alcohol use over 9 mo.: p=0.031 | - | Low: 10, 11, 12  Moderate: 13^C^  High: 9 |
| Bock et al. 1999 [13]  USA | Prospective cohort study | | Homeless individuals  (n=310) | | | - | - | H/R (duration n.r.), SAT  Multivariate: OR (95% CI)  *Completion*   - Foreign birth (ref n.r.): 4.06 (1.78-9.29); p=0.0009 - Age category ≥65y (vs. 0-24y): 5.96 (1.45-24.54); p=0.013 - Jail as enrolment site (vs. Grady Memorial Hospital as enrolment site): 0.26 (0.11-0.63); p=0.002 | Low: 9, 11, 12, 13  Moderate: -  High: 10 |
| Fountain et al. 2005 [14]  USA | Prospective study | | General population (n=3414) | | | - | - | 6H/9H, SAT  Univariate: OR (95% CI)  *Completion*   - Age 50+y (vs. 25-34y): 0.64 (0.50-0.81); p=0.0003 - History of hepatitis A, B or C (vs. no history of liver disease): 1.78 (1.02-3.08); p=0.05 | Low: 9, 12, 13  Moderate: -  High: 10, 11 |
| Goswami et al. 2012 [15]  USA | Prospective cohort study | | General population (initiation: n=496; completion: n=130) | | | 4R/9H, SAT  Multivariable: RR (95% CI)  *Initiation*   - Close contact to a TB case (vs. n.r.): 2.5 (1.8-3.6) - Reasons for screening not related to work (vs. n.r.): 1.6 (1.0-2.5) - Lower educational level (vs. n.r.): 1.3 (1.0-1.6) - Having a regular physician (vs. n.r.): 1.4 (1.0-2.0) - Fear of getting sick with TB without medicine (vs. n.r.): 1.7 (1.2-2.6) - Prior incarceration (vs. n.r.): 1.7 (1.1-2.8) | - | 4R/9H, SAT  Multivariable: RR (95% CI)  *Completion*   - Plan to tell friends or family about LTBI diagnosis (ref n.r.): 2.0 (1.0- 3.9) | Low: 11, 12  Moderate: -  High: 9, 10, 13^A^ |
| Machado et al. 2009 [16]  Brazil | Prospective cohort study | | Case contacts  (n=101) | | | - | - | 6H, SAT  Multivariate: OR (95% CI)  *Non-completion*   - Distance to clinic >10 km (vs. 0-5 km): 0.40 (0.2-0.8); p=0.01 | Low: 11, 12, 13  Moderate: 9  High: 10 |
| Martínez Sanchís et al. 2005 [17]  Spain | Observational study | | Case contacts  (n=458) | | | - | 6H, SAT  Univariate: OR (95% CI)  *Non-adherence*   - Immigrant status (vs. no   immigrant status): 3.42 (1.03-11.04); p=0.02 | - | Low: 9, 12, 13  Moderate: -  High: 10, 11 |
| Minodier et al. 2010 [18]  Canada | Prospective descriptive study | | Immigrants  (initiation: n=645; adherence: n=545) | | | 6H/9H, SAT  Multivariate: OR (95% CI)  *Non-initiation*   - Eastern European origin (vs. South East Asia: 6.91 (1.56-30.75) - Blended family (i.e. living with one of the parents and one parent in law): 3.25 (1.25-8.46) - >2 children under 18y at home: 0.42 (0.17-1.05) - Mean delay between TST and first visit: 1.01 (1.0-1.02) |  | 6H/9H, SAT  Multivariate: OR (95% CI)  *Decreased completion*   - Age >16 years (ref n.r.): 1.82   (1.11-2.99)   - Delay TST-first visit >15 days (ref n.r.): 1.6 (1.12-2.28)   Presence relatives > 18 years in household (ref n.r.): 1.56 (1.0-2.43) | Low: 9, 11, 12, 13  Moderate: -  High: 10 |
| Morano et al. 2013 [19]  USA | Prospective cohort study | | General population (initiation: n=307; completion: n=135) | | | 9H, DOT/SAT  Multivariate: OR (95% CI)  *Initiation*   - Undocumented immigrant (ref n.r.): 3.43 (2.08-5.65); p<0.001 - Born in WHO category 5 country (vs. WHO category 1 country^a^): 14.09 (1.60-123.71); p=0.017 - Born in WHO category 3 country (vs. WHO category 1 country^a^): 2.25 (1.28-3.96); p=0.005 | - | 9H, DOT/SAT  Multivariate: OR (95% CI)  *Completion*   - Not having been incarcerated within 6 months of diagnosis (ref n.r.): 5.95 (1.12-31.62)   - Younger age: 1.03 (1.003-1.06) | Low: 11, 12  Moderate: 9  High: 10, 13 |
| Morisky et al. 2003 [20]  USA | Combination retrospective and prospective chart review | | General population (n=478) | | | - | - | 6H, SAT  Multivariate: OR (95% CI)  *Completion*   - Older age: 0.88 (0.78-0.98); p=0.02 - Hispanic/Latino ethnicity (vs. Asian ethnicity): 0.53 (0.29-0.95); p=0.03 | Low: 9, 11, 12  Moderate: -  High: 10, 13^B^ |
| Narita et al. 2002 [21]  USA | Prospective cohort study | | HIV-infected individuals  (n=228) | | | - | - | 2RZ/12H, DOT, SAT  *Completion*  - RZ (vs. H): p<0.001 | Low: 9, 12, 13  Moderate: -  High: 10, 11 |
| Oni et al. 2012 [22]  South Africa | Prospective study | | HIV-infected individuals  (n=164) | | | - | - | 6H, SAT  Multivariate: OR (95% CI)  *Non-completion*   - Years after HIV diagnosis (by year): 0.81 (0.68-0.98); p=0.03 - Alcohol drinkers (vs. non-drinkers): 4.05 (1.89-9.06); p=0.001 | Low: 11, 12  Moderate: 9  High: 10, 13 |
| Shieh et al. 2006 [23]  USA | Prospective survey | | General population (n=217) | | | - | - | >6H, SAT  Multivariate: OR (95% CI)  *Completion*   - Fear for venepuncture (ref n.r.): 0.43 (0.22-0.85); p=0.015 - Low TB risk perception (ref n.r.): 0.31 (0.13-0.72); p=0.007 | Low: 9, 11, 12  Moderate: -  High: 10, 13^D^ |
| Shukla et al. 2002 [24]  USA | Prospective cohort study | | HCW  (n=388) | | | - | ≥6H, SAT  Multivariate: OR (95% CI)  *Non- adherence (including non-initiation)*   - BCG vaccination (vs. no BCG vaccination): 3.5 (1.8-7.1); p<0.001 - Symptoms on therapy (vs. no symptoms on therapy): 4.5 (2.0-10.1); p=0.001 |  | Low: 9, 11, 12, 13  Moderate: -  High: 10 |
| Trauer et al.2011 [25]  Australia | Prospective study | | Immigrants  (initiation: n=121; completion: n=93) | | | 9H, SAT  Univariate: OR (95% CI)  *Initiation*   - Eastern Mediterranean region of birth (vs. African region of birth): 0.17 (0.06-0.49); p<0.01 | - | 9H, SAT  Univariate: OR (95% CI)  *Completion*   - South-East Asian region of birth (vs. African region of origin: 6.21 (1.59-24.30); p<0.01 - Age 15-34y (vs. age <5y): 0.06 (0.01-0.57); p<0.05 - Age≥35y (vs. age<5-34y): 0.04 (0.00-0.41); p<0.01 | Low: 9, 11, 12, 13  Moderate: -  High: 10 |
| White et al. 2005 [26]  USA | Cohort study | | Inmates  (n=557) | | | - | - | 6H, SAT  Multivariate: OR (95% CI)  *Completion*   - Education (yrs completed): 1.06 (1.01-1.12) - Foreign-born, in USA ≤5 years (vs. born in USA: 0.49 (0.28-0.85) | Low: 9, 11, 12  Moderate: 10  High: 13^C^ |
| Young et al. 2012 [27]  USA | Historical control study | | Immigrants  (n=146) | | | - | - | 9H, SAT  Multivariate: OR (95% CI)  *Completion*   - Younger age at time of CXR: 0.88 (0.8-0.97) - Decreased number of days between positive TST and CXR: 0.999 (0.997- 1.00) - >2 well-child care visits received after the age of 15 months old before TST placement (ref n.r.): 2.69 (1.13- 6.4) | Low: 9, 10, 11, 12, 13  Moderate: -  High: - |
| ***Retrospective observational studies*** | | | | | | | | | |
| Ailinger et al. 1998 [28]  USA | Retrospective review of medical records | | | | Immigrants  (n=65) | - | 6H, SAT  *Adherence*  - Education (years): p=0.034 |  | Low: 18  Moderate: 15, 20^C^  High: 14, 16, 17, 19 |
| Ailinger et al. 2007 [29]  USA | Retrospective review of medical records | | | | Immigrants  (n=129) | - | 9H, SAT | - | Low: 14, 16, 18, 20  Moderate: 19  High: 15, 17 |
| Anger et al. 2012 [30]  USA | Retrospective review of medical records | | | | Case contacts  (n=7597) | 4R/6H/9H, SAT  Multivariate: OR (95% CI)  *Initiation*  - Age (vs. 45-64y)  0-4y: 1.36 (1.30-1.43)  5-17y: 1.31 (.126-1.37)  18-44y: 1.12 (1.08-1.16)  65+y: 0.90 (0.83-0.98)   - Region of birth (vs. non-USA born)   USA-born: 0.95 (0.92-0.99)  Unknown: 0.87 (0.83- 0.91)  - HIV-status (vs. not infected)  Infected: 0.78 (0.61-1.00)  Unknown: 0.90 (0.87-0.92)   - Household exposure (vs. non-household exposure): 1.09 (1.06-1.13) | - | - | Low: 18, 19, 20  Moderate: 14,15  High: 16, 17 |
| Anibarro et al. 2010 [31]  Spain | Retrospective review of medical records | | | | Case contacts  (n=599) | - | - | 2HRZ (+EMB)+2HR/3HR/ 4R/6H/9H, SAT  Multivariate: OR (95% CI)  *Completion*   - Age <36y (vs. ≥36y): 0.33 (0.30- 0.76); p=0.001 - Male (vs. female): 0.58 (0.37-0.92); p=0.02 - Immigrant (<5 years of residence) (vs. not immigrant): 0.21 (0.12-0.37); p<0.001 - Social risk factors present (vs. no social risk factors): 0.21 (0.11- 0.39); p<0.001 | Low: 14, 15, 16, 17, 18, 19  Moderate: 20High: - |
| Cass et al. 2005 [32]  USA | Retrospective database study | | | | General population (n=1582) | - | - | 9H, SAT  Multivariate: OR (95% CI)  *Completion*  (For each factor the opposite was used as reference, except for clinic location)   - Spanish language: 1.75 (1.15-2.67); p<0.05 - Clinic location (vs. Central San Diego)   East County: 0.42 (0.23-0.77); p<0.05  South County: 0.40 (0.22-0.75); p<0.05   - Source case investigation: 2.29 (1.52-3.45); p<0.05 - Treasure Chest intervention (behavioural intervention including self-monitoring and incentives, for children <14 years) : 2.42 (1.66-3.51); p<0.05 - Missed appointment call: 0.31 (0.22-0.45); p<0.05 - Missed appointment letter: 0.35 (0.23-0.52); p<0.05 - Public health nurse referral: 0.29 (0.19-0.45); p<0.05 | Low: 16, 17, 18, 19, 20  Moderate: 14, 15  High: - |
| Chang et al. 2013 [33]  USA | Retrospective review of medical records | | | | General population (n=3918) | - | - | 6H/9H, SAT  Multivariate: OR (95% CI)  *Completion*  - Age (vs. 18- <35y)  <6y: 5.40 (3.19-9.13)  6-<18y: 2.64 (2.10-3.32)   - Referral reason (vs. TST positive from screening)   Correctional/rehabilitation: 0.43 (0.32-0.57)  Postpartum women: 0.64 (0.47-0.87)  - Place of birth (vs. USA)  Asia: 1.62 (1.18-2.22)  Europe: 1.93 (1.06-3.53) | Low: 14, 16, 17, 19, 20  Moderate: 15, 18  High: - |
| Chang et al. 2014 [34]  USA | Retrospective review of medical records | | | | General population (n=1587) | - | - | 9H, SAT  Multivariate: OR (95% CI)  *Non-completion*   - Age 15-18y (vs. 0-5y): 2.0 (1.3-3.0) - Non-Hispanic (vs. Hispanic): 1.8 (1.3-2.4) - Hepatitis (vs. no hepatitis): 24.6 (10.5-62.8) - Symptoms of adverse effects (total symptoms of adverse effects divided by total clinic or home visits during treatment) : 2.0 (1.2-3.0) | Low: 14, 17, 18, 20  Moderate: 15, 16, 19  High: - |
| Codecasa et al. 2013 [35]  Italy | Retrospective review of medical records | | | | General population (n=11963) | - | - | 6H, SAT  Univariate: OR (95% CI)  Only foreign-born multivariate  *Non-completion*   - Foreign-born (vs. Italians): 1.62 (1.45-1.81) - Transaminase increase (ref n.r.): 1.51 (1.27-1.79); p<0.001 - Gastrointestinal problems (ref n.r.): 1.57 (1.26-1.96); p<0.001 - Central nervous system problems (ref n.r.): 1.46 (1.21-1.77); p<0.001 - Dermatological events (ref n.r.): 1.89 (1.39-2.58); p<0.001   Among foreign-born (N=8586)   - Female: 0.81 (0.73-0.89); p<0.001 - Age, years: 1.01 (1.01-1.02); p<0.001 - Adverse events (ref n.r.): 1.33 (1.15-1.53); p<0.05 - Reason to treat (ref unclear):   Access to shelters/rehabilitation:  2.88 (2.43-3.42); p<0.001  Screening in undocumented  subjects:1.20 (1.01-1.44); p<0.05  Among Italians (n=3377)   - Age, years: 1.01 (1.01-1.02); p<0.001 - Adverse events (ref n.r.): 2.70 (2.22-3.28); p<0.001 - HIV-positive (ref n.r.): 5.20 (2.1-12.93);p<0.001 | Low: 17, 18, 19, 20  Moderate: 15, 16  High: 14 |
| Cruz et al. 2012 [36]  USA | Retrospective review of medical records | | | | General population (n=248) | - | - | 6R/9H/9Z+FQ, SAT/ SAT with weekly phone reminders/DOT  Multivariate: OR (95% CI) *Completion*  - DOT (vs. SAT): 7.2 (3.8-13.8) | Low: 16, 17, 18, 19, 20  Moderate: 14, 15  High: - |
| Fresard et al. 2011 [37]  Switzerland | Retrospective review of medical records | | | | General population (n=624) | - | - | 4R/6H, SAT  Multivariate: OR (95% CI)  *Completion*   - R (vs. H): 1.74 (1.11-2.72); p=0.016 | Low: 14, 16, 17, 18,19  Moderate: 15, 20^E, G^  High: - |
| Gershon et al. 2004 [38]  Canada | Retrospective review of medical records | | | | General population (n=308) | According to Canadian Tuberculosis Standards, 5th edition  Multivariate: OR (95% CI)  *Initiation*   - HCW (vs. n.r.): 0.55 (0.32-0.93); p=0.03 - Age (per 10-y interval): 0.54 (0.43-0.67); p<0.0001 - Recent contact with active TB and/or TB skin test conversion (vs. n.r.): 1.74 (1.01-2.98); p=0.04 - Previous BCG vaccination (vs. n.r.): 0.50 (0.30-0.84); p=0.09 - Abnormal CXR findings consistent with previous TB (vs. n.r.): 2.40 (1.29-4.48); p=0.06 | - | - | Low: 16, 17, 18, 19, 20  Moderate: -  High: 14, 15 |
| Gilroy et al. 2000 [39]  USA | Retrospective review of medical records | | | | General population (initiation: n=510; completion: n=500) | 6H, SAT  *Initiation*  - Older age: p=0.000002  *Non-initiation*  - Men: p=0.0307  - Alcohol use: p=0.00001  Ethnicity p=0.00009  Initiated vs. did not initiate (%):  White 46.6 vs. 34.3  Black 31.3 vs. 50.9  Other 22.1 vs. 14.9 | - | 6H, SAT  *Non-completion*   - Use of alcohol by men (vs. did not use alcohol): p=0.001 - Use of concomitant medications by women (vs. no use of concomitant medication): p=0.005 - Elevated ALT levels (vs. normal ALT levels): p<0.001 | Low: 14, 18, 19, 20  Moderate: 15  High: 16, 17 |
| Grinsdale et al. 2011 [40]  USA | Retrospective review of medical records | | | | Case contacts  (initiation: n=261; completion: n=205) | 4HR/6H/9H, SAT  Multivariate: OR (95% CI)  *Initiation*   - Positive QFT-G (vs. positive TST): 5.18 (2.1-14.18) | - | 4HR/6H/9H, SAT  Multivariate: OR (95% CI)  *Completion among all infected contacts*   - Positive QFT-G+ (vs. positive TST): 3.37 (1.78-6.56) | Low: 16, 17, 18, 19, 20  Moderate: 15, *20^H^ (Only for determinants on completion)*  High: 14 |
| Haley et al. 2008 [41]  USA | Retrospective review of medical records | | | | General population (n=749) | - | - | 4R, SAT  Univariate: RR (95% CI)  Multivariate: OR (95% CI)  *Non-completion*  Univariate analysis   - Hispanic ethnicity (vs. Non-Hispanic): 0.6 (0.4-0.7)   Multivariate analysis, Hispanic subjects (n=534)   - Contact with an infectious TB case (vs. no contact): 3.7 (1.8-7.4); p<0.001 - Alcohol use reported at baseline (vs. none reported): 1.7 (1.1-2.8); p=0.02 - Other medications reported at baseline (vs. none reported): 2.2 (1.3-3.8); p=0.01   Multivariate analysis, non-Hispanic subjects (n=215)   - Black race (ref unclear): 2.6 (1.5-4.7); p=0.001 - Age (per year): 0.97 (0.94-0.99); p=0.03 - Foreign birth (vs. USA birth): 0.5 (0.2- 0.9); p=0.02 | Low: 14, 16, 17, 18, 19, 20  Moderate: 15  High: - |
| Hirsch-Moverman et al. 2010 [42]  USA | Retrospective review of medical records | | | | General population (n=312) | - | - | 6H/9H/12H, SAT  Multivariate: OR (95% CI)  *Completion*   - Foreign-born (vs. USA-born): 0.55 (0.30-1.00); p=0.0495 - Currently homeless (vs. not currently homeless): 0.43 (0.21-0.89); p=0.022 - Current alcohol use (vs. no current alcohol use): 0.51 (0.28-0.91); p=0.024 | Low: 16, 17, 18, 19  Moderate: 15, 20^E^  High: 14 |
| Horsburgh et al. 2010 [43]  Canada, USA | Retrospective review of medical records | | | | General population (initiation: n=720; completion: n=1959) | 2RZ/4R/6H/9H, SAT  Multivariate: OR (95% CI)  *Non-initiation*   - Employee of a health-care facility (vs. no/unknown): 4.74 (1.75-12.9); p=0.003 - Contact with a person with TB (vs. no/unknown): 0.19 (0.07-0.50); p=0.001 | - | 2RZ/4R/6H/9H, SAT  Multivariate: OR (95% CI)  *Non-completion*   - Resident in a congregate setting (vs. never or unknown): 2.94 (1.58-5.56); p=0.001 - 9-mo H (vs. other regimens): 2.08 (1.23-3.57); p=0.008 - IDU (vs. no/unknown): 2.13 (1.04-4.35); p=0.038 - Employee of a health-care facility (vs. no/unknown): 1.37 (1.00-1.85); p=0.049 - Age ≥15y (vs. <15y): 1.45 (1.14-1.94); p=0.004 | Low: 16, 17, 18, 19  Moderate: 15, 20  High: 14 |
| Kan et al. 2013 [44]  Sweden | Retrospective review of medical records | | | | General population (n=360) | - | - | 9H/other (24%)^b^, SAT  Multivariate: OR (95% CI)  *Non-completion*   - Cause of screening/referral (vs. anti-TNF-α candidates)   Contacts: 13.5 (1.6-116.7); p=0.006  Asylum seekers: 19.8 (1.7-232.1);  p=0.002  Among contacts   - Somali origin (vs. non-Somali origin): 2.0 (1.03-3.9); p=0.041   Among female   - Somali origin (vs. non-Somali origin): 2.9 (1.5-5.7); p=0.0025 | Low: 16, 17, 18, 19, 20  Moderate: 15  High: 14 |
| Kwara et al. 2008 [45]  USA | Retrospective review of medical records | | | | General population (initiation: n=845; completion: n=690) | 9H, SAT  Multivariate: OR (95% CI)  *Non-initiation*   - Pregnant (vs. not pregnant): p<0.0001 | - | 9H, SAT  Multivariate: OR (95% CI)  *Non-completion*   - No medical insurance (vs. medical insurance): 1.7 (1.1-2.7); p=0.023 - Reported side effects (vs. no reported side effect): 3.6 (2.2-6.2); p<0.0001 | Low: 16, 17, 18, 19, 20  Moderate: 14, 15  High: - |
| Lardizabal et al. 2006 [46]  USA | Retrospective review of medical records | | | | General population (n=474) | - | - | 4R/9H, SAT  Multivariate: OR (95% CI)  *Completion*   - R (vs. H): 5.10 (3.26-8.14); p<0.0001   *Non-completion* (univariate)  - Employment (vs. n.r.): p=0.03 | Low: 14, 16, 17, 18, 19, 20  Moderate: 15  High: - |
| Lee et al. 2002 [47]  USA | Retrospective review of medical records | | | | General population (n=148) | - | - | 2RZ, SAT  *Non-completion*   - Illicit drugs use reported (vs. no illicit drug use reported): p<0.05 - White or Asian race/ethnicity (vs. Hispanics): p<0.05 - Presumed non-recent TB infection (vs. presumed recent TB infection): p<0.05 | Low: 14, 18, 19, 20  Moderate: 16, 17  High: 15 |
| Li et al. 2010 [48]  USA | Retrospective review of medical records | | | | General population (n=15035) | - | - | 4R/4RFB/6H/9H, SAT  Multivariate: RR (95% CI)  *Completion*   - Age ≥35y (vs. 25-34y): 1.16 (1.11-1.22) - Ethnicity (vs. non-Hispanic white)   Asian: 1.20 (1.10-1.30)  Non-Hispanic black: 1.11 (1.02-  1.20)  Hispanic: 1.10 (1.02-1.19)   - Non-USA-born (vs. USA-born): 1.08 (1.03-1.13) - Risk group (vs. low risk)   Contact: 1.51 (1.38-1.66)  Medical risk: 1.45 (1.32-1.60)  Population risk: 1.16 (1.07-1.27)   - Ever on DOT (vs. never on DOT): 1.26 (1.18-1.34) - Rifamycin alone (vs. H alone): 1.20 (1.14-1.26) | Low: 14, 16, 17, 18, 19, 20  Moderate: 15  High: - |
| Lobato et al. 2005 [49]  USA | Retrospective review of medical records | | | | Inmates and homeless (n=1211) | - | - | 2RZ, SAT/DOT  Multivariate: OR (95% CI)  *Non-completion*   - Female: 0.35 (0.23-0.54); p<0.001 - Hispanic (ref unclear): 0.59 (0.46-0.75); p<0.001 - Unemployed (ref n.r.): 1.43 (1.07-1.90); p=0.02 - IDU (ref n.r.): 0.54 (0.31-0.95); p=0.03 - Excess alcohol (ref n.r.): 1.35 (1.04-1.76); p=0.03 | Low: 14, 15, 16, 17, 18, 19, 20  Moderate: -  High: - |
| LoBue et al. 2003 [50]  USA | Retrospective review of medical records | | | | General population (n=3788) | - | - | 6H/9H, SAT  Multivariate: OR (95% CI)  *Completion*   - Female: 1.2 (1.0-1.4); p=0.03 - Age (vs. 65+y)   0-14y: 4.1 (2.2-7.8); p<0.01  15-34y: 2.2 (1.1-3.9); p=0.03   - White Hispanic (vs. Black, non-Hispanic): 1.5 (1.0-2.3); p=0.04 - Country of birth non-USA (vs. USA): 1.4 (1.1-1.7); p<0.01 - Excess alcohol (vs. no excess alcohol): 0.1 (0.0-0.6); p<0.01 - Homeless (vs. not homeless): 0.2 (0.1-0.5); p<0.01 - Adverse effects other than hepatotoxicity (vs. no other adverse effects): 0.8 (0.7-0.9); p=0.03 | Low: 14, 16, 17, 18, 19, 20  Moderate: 15  High: - |
| Lopez et al. 2011 [51]  Spain | Retrospective review of medical records | | | | Inmates  (n=810) | - | - | 2RZ/3HR/4R/9H, DOT  OR (95% CI)  *Non-completion*   - 9 mo. H (vs. short courses): 1.56 (1.14-2.12); p=0.006 - RZ (vs. HR): 2.11 (1.09-4.09); p=0.029 - HIV-positive (vs. HIV-negative): 2.11 (1.34-3.33); p=0.002   *Voluntary non-completion for no apparent reason*   - 9 mo. H (vs. short courses): 2.03 (1.30-3.15); p=0.002   *Non-completion due to transfer or release*   - 9 mo. H (vs. short courses): 30.22 (4.07-224.29); p<0.0001   *Non-completion due to hepatotoxicity*   - 9 mo. H (vs. short courses): 2.94 (1.05-8.24); p=0.037   *Non-completion due to skin rash*   - RZ (vs. 9 mo. H): 70.07 (95% CI n.a.); p<0.0001 - RZ (vs. HR): 6.96 (95% CI n.a.); p=0.024 | Low: 14, 15, 17, 18, 19  Moderate: 20^I^  High: 16 |
| Marks et al. 2000 [52]  USA | Retrospective review of medical records | | | | Case contacts  (n=1725) | 6H/12H/R, SAT/DOT  Multivariate: RR (95% CI)  *Initiation*   - Age, 6-14y (ref unclear): 1.3 (1.1-1.5) - Age, 45-64y (ref unclear): 0.9 (0.8-1.0) - Foreign born (vs. USA-born): 1.2 (1.1-1.3) | - | - | Low: 16, 17, 18  Moderate: 15, 19, 20^B^  High: 14 |
| McElroy et al. 2005 [53]  USA | Retrospective review of medical records | | | | General population (n=8087) | - | - | 2RZ, SAT  *Completion*   - Twice weekly (vs. daily RZ): p<0.001 | Low: 19  Moderate: 14, 15, 18, 20^B^  High: 16, 17 |
| Mindachew et al. 2011 [54]  Ethiopia | Cross-sectional study | | | | HIV-infected individuals  (n=319) | - | 6H, SAT  Multivariate: OR (95% CI)  *Adherence*   - Received explanation about treatment (vs. did not receive explanation about treatment): 7.74 (3.14-19.06); p<0.001 - H related adverse effects (vs. n.r.): 0.06 (0.02-0.18); p<0.001 - Good feeling/comfortable to take H in front of others (vs. n.r.): 5.98 (2.31-15.50); p<0.001 - Attending clinical appointment regularly (vs. those who did not attend regularly): 4.00 (1.06-15.07); p=0.04 | - | Low: 14, 16, 17, 20  Moderate: 19  High: 15, 18 |
| Nuzzo et al. 2013 [55]  USA | Retrospective review of medical records | | | | General population (initiation: n=841; completion: n=652) | 4R/9H, SAT  Multivariate: OR (95% CI)  *Initiation*   - Female: 0.79 (0.63-1.0); p=0.049 - Age 0-14y (vs. 25-44y): 2.5 (1.62-3.85); p<0.001 - Refugee (vs. USA- Born): 4.8 (3.1-7.5); p<0.001 | - | 4R/9H, SAT  *Completion*  - Status (vs. USA-born)  Refugee: 7.2 (4.2-12); p<0.001  Non-refugee foreign-born: 2.8 (1.6-4.9); p<0.001 | Low: 16, 17, 18, 19, 20  Moderate: 15  High: 14 |
| Page et al. 2006 [56]  USA | Retrospective review of medical records | | | | General population (n=2149) | - | - | 4R/9H, SAT/DOT  Multivariate: OR (95% CI)  *Completion*   - R (vs. H): 2.88 (2.27-3.66) - Region of origin (vs. USA, Canada, Europe)   Latin America and Caribbean: 1.68 (1.21-2.33)  Asia and other: 2.25 (1.44-3.53)   - Age 18-35y (vs. <18y): 0.48 (0.33-0.71) - Adverse drug reaction (vs. no adverse drug reactions)   Hepatotoxicity: 0.003 (0.00-0.27)  Other: 0.21 (0.15-0.30) | Low: 16, 17, 18, 19, 20  Moderate: 14, 15  High: - |
| Parsyan et al. 2007 [57]  USA | Retrospective review of medical records | | | | General population (n=1572) | - | - | 6H/9H, SAT  Multivariate: OR (95% CI)  *Non-completion (aged <35y)*   - Country of birth (vs. other countries)   Haiti: 2.17 (1.49-3.17); p<0.0001  Dominican Republic: 1.93 (1.08-3.43); p=0.026  China with Hong Kong: 0.53 (0.38-0.72); p<0.0001  Vietnam: 0.45 (0.31-0.65);  p<0.0001 | Low: 16, 17, 18, 19, 20  Moderate: 15  High: 14 |
| Priest et al. 2004 [58]  USA | Retrospective review of medical records | | | | Immigrants  (n=423) | - | - | 2RZ, DOT  Multivariate: OR (95% CI)  *Non-completion*   - Age (per year): 1.08 (1.03-1.13); p<0.001 - Development of clinical symptoms (ref n.r.): 9.73 (5.12-18.51); p<0.001 | Low: 14, 15, 16, 17, 18, 19, 20  Moderate: -  High: - |
| Rennie et al. 2007 [59]  UK | Retrospective review of medical records | | | | General population (n=591) | - | - | 3HR/6H, SAT  Multivariate: OR (95% CI)  *Non-completion*   - Age (continuous): 1.04 (1.02-1.06) - Clinic attendance before treatment (vs. clinic non-attendance before treatment): 0.54 (0.37-0.78) - Regimen choice offered (vs. no regimen choice offered): 0.43 (0.30-0.60) | Low: 15, 16, 17, 19, 20  Moderate: 14, 18  High: - |
| Vinnard et al. 2013 [60]  USA | Retrospective review of medical records | | | | General population (n=219) | - | - | 9H, SAT  Multivariate: HR (95% CI)  *Non-completion due to suspected toxicity*   - Hepatitis C (ref n.r.): 3.03 (1.08-8.52) | Low: 16, 17, 18, 19  Moderate: 14, 15, 20^F^  High: - |
| White et al. 2003 [61]  USA | Retrospective review of medical records | | | | General population (n=1079) | - | - | 6H, SAT/DOT  Multivariate: OR (95% CI)  *Completion*  Multivariate analysis   - DOT (vs. SAT): 1.93 (1.25-3.00); p=0.003 - Male: 0.67 (0.49-0.90); p=0.009 - Age (each additional year): 1.02 (1.01-1.03); p=0.001 - Asian/Pacific Islander (vs. white): 1.89 (1.17-3.03); p=0.009 - 1997-1998 cohort (vs. 1993-June 1994 cohort): 1.49 (1.12-1.99); p=0.006 | Low: 15, 16, 17, 18, 19, 20  Moderate: 14  High: - |
| Young et al. 2009 [62]  USA | Retrospective review of medical records | | | | General population (n=777) | - | - | 4R/9H, SAT  Multivariate: OR (95% CI)  *Completion*   - R (vs. H): 4.40 (1.948-9.917); p<0.001 - Increasing age: 1.02 (1.006-1.042); p=0.009 - Asian (vs. white): 8.59 (2.223-33.224); p=0.002 | Low: 16, 17, 18, 20  Moderate: 15, 19  High: 14 |

ALT: alanine transaminase; BCG: Bacillus Calmette-Guérin; CI: confidence interval; CXR: chest radiograph; DOT: directly observed therapy; EMB: ethambutol; FQ: fluoroquinolone; H: isoniazid; HCW: healthcare worker; HIV: human immunodeficiency virus; HR: hazard ratio; IDU: injecting drug user; H: isoniazid; km: kilometre; LTBI: latent tuberculosis infection; mo.: month; n: number; n.a.: not available; n.r.: not reported; OR: odds ratio; PWID: people who inject drugs; QFT-G: QuantiFERON®-TB Gold; QFT-GIT: QuantiFERON-TB Gold In-Tube; R: rifampicin; RCT: randomised controlled trial; RFB: rifabutin; RR: relative risk; RZ: rifampicin and pyrazinamide; SAT: self-administered therapy; TB: tuberculosis; TST: tuberculin skin test; UK: United Kingdom; USA: United States of America; vs.: versus; WHO: World Health Organisation; y: year; Z: pyrazinamide.
Quality aspects of randomised controlled trials: 1: Randomisation; 2: Allocation concealment; 3: Blinding; 4: Similarity treatment and control group; 5: Intention-to-treat analysis; 6: Drop-outs (only applicable for studies presenting determinants of adherence or completion); 7: Treatment adherence assessment; 8: Other bias. Quality aspects of prospective observational studies: 9: Drop-outs (only applicable for studies presenting determinants of adherence or completion); 10: Treatment adherence assessment; 11: Confounders; 12: Confidence intervals; 13 Other bias. Quality aspects of retrospective observational studies: 14: Drop-outs (only applicable for studies presenting determinants of adherence or completion); 15: Treatment adherence assessment; 16: Confounders; 17: Confidence intervals; 18: Recall; 19: Retrospective selection; 20: Other bias.

A: Limited power; B: Limited population characteristics; C: Selection bias; C: Small sample size; D: Completion not well defined; E: Two time periods: inconsistency in recording can be occurred; F: Unknown how many Individuals with LTBI did not have a HIV or hepatitis C test; G: Analysis not clear of group who switched treatment regimen; H: Type of treatment not taken into account in model on determinants of completion; I: Treatment was indicated as primary chemoprophylaxis for a subgroup (n=40, initiated=20), subgroup included in analysis. Treatment description changed during years; J: outcome data only available for subgroup.
a: WHO defined 5 categories of TB prevalence based on 1^st^ (least prevalent) to 5^th^ (most prevalent); b: H 6 mo., R 4-6 mo., HR ≥3 mo., RZ 2 mo., other combinations.
If only p-values are presented, these represent p-values from univariate risk factor analyses.
