# Supplementary material for: Interventions for improving adherence to treatment for latent tuberculosis infection: a systematic review
Source: BMC Infect Dis. 2016 Jun 8;16:257. doi: 10.1186/s12879-016-1549-4 (PMC4897858; doi:10.1186/s12879-016-1549-4)
Supplement: Additional file 4: — Study characteristics, outcomes, and risk of bias assessment of articles on interventions to improve initiation, adherence and completion of LTBI treatment regimens. (DOCX 104 kb) [file 12879_2016_1549_MOESM4_ESM.docx]

## Additional file 4– Study characteristics, outcomes, and risk of bias assessment of articles on interventions to improve initiation, adherence and completion of LTBI treatment regimens.

| **Reference, country** | **Study design** | **Study population**  **(sample size)** | **Treatment (if intervention other than treatment)** | | **Results per intervention** | **Risk of bias assessment of each study** |
| --- | --- | --- | --- | --- | --- | --- |
|  |  |  | **Description intervention** | **Description control group** |  |  |
| ***Randomised controlled trial*** | | | | | | |
| *Intervention: short treatment regimen* | | | | | | |
| Jasmer et al. 2002 [5]  USA | Multicenter, prospective open-label clinical trial | General population (n=589) | I - 2RZ  - n=307 | II - 6H  - n=282 | Completion  - I 61%  - II 57%  Univariate OR (95%CI)  -1.19 (0.84-1.64) | Low: 5, 8  Moderate: 2, 3, 6  High: 1, 4, 7 |
| Jiménez-Fuentez et al. 2013 [6]  Spain | RCT | Immigrants  (n=590) | I - 3HR  - n=296 | II - 6H  - n=294 | Completion  - I 72%  - II 52%  Poor completion  Univariate OR (95% CI)  - II vs. I 2.45 (1.68-3.57); p=0.0001 | Low: 1, 6, 5 (completion rate), 7  Moderate: 2, 3  High: 4, 5 (OR), 8^A^ |
| Menzies et al. 2004 [63]  Canada | Open-label RCT | General population (n=116) | I - 4R  - n=58 | II - 9H  - n=58 | Completion  - I 91%  - II 76%  Univariate: RR (95% CI)  - 1.2 (1.02-1.4) | Low: 1, 4, 5, 6, 7  Moderate: 2  High: 3, 8^B^ |
| Menzies et al. 2008 [64]  Canada, Saudi Arabia, Brazil | RCT | General population (n=847) | I - 4R  - n=420 | II - 9H  - n=427 | Completion  - I 78%  - II 60%  - p<0.001 | Low: 1, 4, 5, 6, 7, 8  Moderate: 2  High: 3 |
| Spyridis et al. 2007 [65] Greece | RCT | General population  (n=926) | I - Period 1: 1995-1998  - 4HR  - n=238  II - Period 2: 1999-2002  - 4HR  - n=236 | III - Period 2: 1999-2002  - 3HR  - n=220  IV - Period 1: 1995-1998  - 9H  - n=232 | Excellent/moderate compliance rate  - I 92%  - IV 86%  - p=0.011  - II 94%  - III 95%  - p=0.510 | Low: 1, 4, 5, 6, 7, 8  Moderate: 2  High: 3 |
| Sterling et al. 2011 [66]  Brazil, Canada, Spain, USA | Prospective open-label randomised non-inferiority trial | Case contacts  (n=7731) | I - 3H+RPT, DOT  - n=3986 | II - 9H, SAT  - n=3745 | Completion rate  - I 82%  - II 69%  - p<0.001 | Low: 1, 4, 5, 6  Moderate: 2  High: 3, 7, 8^C^ |
| Tortajada et al. 2005 [67]  Spain | Multicenter randomised, comparative and prospective trial | Case contacts  (n=352) | I - 2RZ, SAT  - n=153 | II - 6H, SAT  - n=199 | Daily adherence rate  - I 82%  - II 77%  - p=0.21  Completion rate  - I 70%  - II 73%  - p=0.73  Discontinued treatment rate  - I 20%  - II 9%  - p=0.005 | Low: 1, 5, 6, 7, 8  Moderate: 2  High: 3, 4 |
| *Intervention: DOT* | | | | | |  |
| Batki et al. 2002 [2]  USA | RCT | PWID  (n=111) | 6H | | Completion rate (95% CI) - I 60% (44-75) - II 77% (61-91) - III 13% (3-23)  - I, II vs. III: p<0.0001 | Low: 1, 2, 5, 6  Moderate: -  High: 3, 4, 7, 8^B, C^ |
|  |  |  | I - Standard care with methadone treatment (MT)  - DOT  - Substance abuse counselling  - n=37  II - Standard care with MT  - DOT  - No counselling  - n=35 | III - Standard care without MT  - SAT  - No counselling  - n=39 |  |  |
| Chaisson et al. 2001 [68]  USA | Factorial designed RCT | PWID  (n=300) | 6H | | Completion rate  - I 80%  - II 78%  - III 79%  - I vs. III p=0.86  - I vs. II p=0.73 | Low: 1, 4, 5, 6, 7, 8  Moderate: 2  High: 3 |
|  |  |  | I - Supervised group: assigned to  an outreach nurse  - Twice weekly DOT  - n=99  II - Peer group: monthly supply of medication  - Daily SAT  - n=101 | III - Routine group: monthly  supply of medication  - Daily SAT  - n=100 |  |  |
| Matteelli et al. 2000 [69]  Italy | Multicenter, prospective randomised open-label study | Immigrants  (n=208) | 6H | | 6H, SAT/DOT  Completion  - I 7.3%  - II 26%  - III 41%   - I vs. II p=0.001 - I vs. III p=0.006 | Low: 1, 4, 5, 6, 7, 8  Moderate: 2  High: 3 |
|  |  |  | I - DOT  - n=82 | II - SAT (twice weekly)  - n=73  III - SAT (daily)  - n=53 |  |  |
| Sterling et al. 2011 [66]  Brazil, Canada, Spain, USA | Prospective open-label randomised non-inferiority trial | Case contacts  (n=7731) | I - DOT, 3H+RPT  - n=3986 | II - SAT, 9H  - n=3745 | Completion rate  - I 82%  - II 69%  - p<0.001 | Low: 1, 4, 5, 6  Moderate: 2  High: 3, 7, 8^C^ |
| *Intervention: incentive* | | | | | |  |
| Batki et al. 2002 [2]  USA | RCT | PWID  (n=111) | 6H | | Completion rate (95% CI) - I 60% (44-75) - II 77% (61-91) - III 13% (3-23)  - I, II vs. III: p<0.0001 | Low: 1, 2, 5, 6  Moderate: -  High: 3, 4, 7, 8^B, C^ |
|  |  |  | I - Usual care with methadone treatment (MT)  - DOT  - Substance abuse counselling  - n=37  II - Usual care with MT  - DOT  - No counselling  - n=35 | III - Usual care without MT  - SAT  - No counselling  - n=39 |  |  |
| Malotte et al. 2001 [8]  USA | RCT | PWID  (n=163) | 6H/12H, DOT | | Completion rate  - I 53%  - II 60%  - III 4%  Multivariate OR (95% CI)  - I vs. III 29.7 (6.4-137.5) - II vs. III 45.5 (9.7-214.6) | Low: 2, 4, 5, 7  Moderate: 1, 3, 6, 8^B^  High: - |
|  |  |  | I - Twice weekly DOT at location chosen by participant  - Monetary incentive ($5)  - n=53  II - Twice weekly DOT at study community site  - Monetary incentive ($5)  - n=55 | III - As condition I  - No incentive  - n=55 |  |  |
| Tulsky et al. 2004 [11]  USA | Prospective, randomised clinical trial | Homeless individuals  (n=119) | 4HR/6H, DOT/SAT | | Completion rate  - I 85%  - II 77% | Low: 1, 2, 5 (completion rates) 6  Moderate: 3, 4  High: 5 (p-value), 7, 8^B, C^ |
|  |  |  | I - Cash incentive: a $5 cash  payment  - n=65 | II - Non-cash incentive: coupons, phone cards or bus cards, each with a value of $5  - n=54 |  |  |
| White et al. 2002 [70]  USA | RCT | Inmates (after release)  (n=216) | 6H, DOT | | Completion rate  - I 12%  - II 12%  Multivariate OR (95% CI):  - 1.07 (0.47-2.40) | Low: 1, 2, 4, 5, 6, 7  Moderate: 3  High: 8 |
|  |  |  | I - Promise of incentive, $25  equivalent in food or  transportation vouchers  - n=113 | II - No education  - No incentives  - n=103 |  |  |
| *Intervention: social interventions* | | | | | |  |
| Chaisson et al. 2001 [68]  USA | Factorial designed RCT | PWID  (n=300) | 6H | | Completion rate  - I 80%  - II 78%  - III 79%  - I vs. III p=0.86  - I vs. II p=0.73 | Low: 1, 4, 5, 6, 7, 8  Moderate: 2  High: 3 |
|  |  |  | I - Supervised group: assigned to an outreach nurse  - Twice weekly DOT  - n=99  II - Peer group: monthly supply of medication  - Daily SAT  - n=101 | III - Routine group: monthly  supply of medication  - Daily SAT  - n=100 |  |  |
| Hirsch-Moverman et al. 2013 [3]  USA | RCT | General population  (n=250) | 9H, SAT | | Completion rate  - I 61%  - II 57%  Multivariate RR (95% CI):  - 1.096 (0.850-1.414) | Low: 1, 4, 5, 6, 7, 8  Moderate: 2, 3  High: - |
|  |  |  | I - Peer-based intervention.  Utilising health belief model, social learning theory, and precaution adoption process model, enriched by social support concepts  - Compensation for time and travel  - n=128 | II - Usual care  - Compensation for time and travel  - n=122 |  |  |
| Hovell et al. 2003 [71]  USA (Am J Public Health) | RCT | General population  (n=286) | 6H/9H, SAT | | Completion rate  - I 51%  - II 42%  - III 38%  - p>0.05 between I, II and/or III  Pills taken  - I significantly more compared to II & III: p<0.05 | Low: 5, 7, 8  Moderate: 1, 2, 3, 4, 6  High: - |
|  |  |  | I - Adherence coaching  - n=92  II - Self-esteem counselling: attention control  - n=98 | III - Usual care  - n=96 |  |  |
| Kominski et al. 2007 [7]  USA | RCT | General population  (n=794) | ≥6H, SAT | | Completion rate - I 75%  - II 74%  - III 84%  - IV 76%  - III vs. IV: p=0.051 | Low: 1, 4, 8  Moderate: 2, 3, 5, 6  High: 7 |
|  |  |  | I - Peer counsellor  - n=199  II - Contingency contracting (reward negotiated between parents and adolescent in exchange for the adolescent’s compliant behaviour and completion of care)  - n=203  III - Combined peer counsellor/contingency contracting  - n=197 | IV - Usual care  - n=195 |  |  |
| Nyamathi et al. 2006 [9]  USA | Two-group site-randomised design | Homeless individuals  (n=520) | 6H, DOT | | Completion rate - I 62%  - II 39%  Multivariate OR (95% CI):  - 3.01 (2.15-4.20) | Low: 1, 5, 6, 7  Moderate: 2  High: 3, 4, 8 |
|  |  |  | I - Nurse management  • Changing context activities  • TB and HIV risk reduction education  • Training in coping, self- management, and communication skills  • Training in social and cognitive problem solving  • Develop relationships and social networks  - Tracked when missing DOT, escorted to service appointments  - Incentives  - n=279 | II - Usual care  - Incentives  - n=241 |  |  |
| White et al. 2002 [70]  USA | RCT | Inmates  (n=209) | 6H, DOT | | Completion rate  - I 23%  - II 12%  Multivariate OR (95% CI):  - 2.2 (1.04-4.72) | Low: 1, 2, 4, 5, 6, 7  Moderate: 3  High: 8 |
|  |  |  | I - Education every 2 weeks  while in jail  - n=106 | II - No education  - No incentives  - n=103 |  |  |
| ***Prospective observational studies*** | | | | | |  |
| *Intervention: social intervention* | | | | | |  |
| Ailinger et al. 2010 [72]  USA | Pre-experimental design | Immigrants  (n=184) | 9H, SAT | | Adherence:  - I 157 pills taken (5.2 months)  - II 129 pills taken (4.3 months)  - p=0.028 | Low: 9, 13  Moderate: 11  High: 10, 12, 14 |
|  |  |  | I - Usual care  - Cultural intervention: based on Latino cultural values, 5 components  - n=53 | II - Usual care (historical  sample)  - n=131 |  |  |
| Goldberg et al. 2004 [73]  USA | Cohort study | Immigrants  (n=946) | 6H/9H, SAT | | Initiation rate I vs. II:  - Overall: 88% vs. 73%  - Former Soviet Union: 73% vs.  57% (p=0.007)  - Former Yugoslavia: 99% vs. 39%  Completion rate I vs. II.:  - Overall: 82% vs. 37%  - Former Soviet Union: 76% vs.  45%  - Former Yugoslavia: 94% vs. 60%  - Somalia: 88% vs. 34%  - Other: 63% vs. 31%  All outcomes had p<0.001, unless stated otherwise | Low: 13, 14  Moderate: 9, 11  High: 10, 12 |
|  |  |  | I - Cultural case management program: home readings of TST, culturally appropriate  education, referral health and social service needs  - n=389 | II - Traditional approach  (historical sample 1996-  1998 of refugees)  - n=557 |  |  |
| *Intervention: other* | | | | | |  |
| Sahni et al. 2009 [74]  USA | Before-and-after design | HCW  (n=107) | H (duration n.r.), SAT | | Initiation rate  - I 52%  - II 11%  Univariate OR (95% CI):  - 8.8 (3.1-23) | Low: 12  Moderate: 9, 13  High: 10, 11, 14^B, D^ |
|  |  |  | I - HCWs with positive IGRA,  hired from July 1, 2007  - n=62 | II - HCWs with LTBI identified by TST from January 1 through June 30, 2007  - n=45 |  |  |
| ***Retrospective observational studies*** | | | | | |  |
| *Intervention: treatment* | | | | | |  |
| White et al. 2003 [61]  USA | Retrospective review of medical records | General population  (n=1079) | I - 6H, DOT  - Incentives^a^ (lunch, coupon for fast-food restaurant, bus coupon)  - n=145 | II - 6H, SAT  - n=934 | Completion rate  - I 70%  - II 48%  Multivariate: OR (95% CI)  - 1.93 (1.25-3.00) | Low: 16, 17, 18, 19, 20, 21, 22  Moderate: 15  High: - |
| *Intervention: incentives* | | | | | |  |
| Cass et al. 2005 [32]  USA | Retrospective database study | General population  (n=1582) | 9H, SAT | | Completion rate  - I 92%  - II 82%  Multivariate: OR (95% CI)  - 2.42 (1.66-3.51) | Low: 17, 18, 20, 21, 22  Moderate: 15, 16  High: 19 |
|  |  |  | I - Behaviour modification  techniques of self-monitoring (calendar and stickers) and incentives (stuffed animal or toy)  - n=741 | II - Historical group,  intervention not yet  implemented  - n=841 |  |  |
| *Intervention: other* | | | | | |  |
| Chang et al. 2013 [33]  USA | Retrospective review of medical records | General population  (n=3918) | 6H/9H, SAT | | Completion rate  - I 90%  - II 73%  Multivariate: OR (95% CI)  -2.94 (2.33-3.71)  Multivariate: RR (95% CI)  - 1.21 (1.18-1.24) | Low: 15, 17, 18, 21, 22  Moderate: 16, 20  High: 19 |
|  |  |  | I - Monthly home follow-up of  individuals with increased risk for non-completion or high TB-risk individuals^b^  - n=986 | II - Clinical follow-up  - n=2932 |  |  |

CI: confidence interval; CXR: chest X-ray; DOT: directly observed therapy; H: isoniazid; HCW: healthcare worker; IGRA: Interferon Gamma Release Assay; LTBI: latent tuberculosis infection; MT: methadone treatment; n: number; OR: odds ratio; PWID: people who inject drug; R: rifampicin; RZ: rifampicin and pyrazinamide; RCT: randomised controlled trial; RPT: rifapentin; RR: relative risk; SAT: self-administered therapy; TB: tuberculosis; TST: tuberculin skin test; USA: United States of America; vs.: versus; y: years.

Quality aspects of randomised controlled trials: 1: Randomisation; 2: Allocation concealment; 3: Blinding; 4: Similarity treatment and control group; 5: Intention-to-treat analysis; 6: Drop-out; 7: Treatment adherence assessment; 8: Other bias; Quality aspects of prospective observational studies: 9: Drop-outs (only applicable for studies presenting determinants of adherence or completion); 10: Treatment adherence assessment; 11: Confounders; 12: Confidence intervals; 13: Comparability of groups; 14: Other bias. Quality aspects of retrospective observational studies: 15: Drop-outs (only applicable for studies presenting determinants of adherence or completion); 16: Treatment adherence assessment; 17: Confounders; 18: Confidence intervals; 19: Comparability of groups; 20: Recall; 21: Retrospective selection; 22: Other bias.

A: Limited power; B: Small sample size; C: Exposure bias; D: outcomes assessed with a telephone survey.
a: Patient at risk for progression to TB received additional incentives. b: Individuals with increased risk or non-completion or high TB-risk individuals: children <6y, contacts to TB cases, and TST converters).

**References**

1. Bastos ML, Menzies D, Belo MT, et al. Changes in QuantiFERON(R)-TB Gold In-Tube results during treatment for tuberculous infection. Int J Tuberc Lung Dis **2013**; 17:909-16.

2. Batki SL, Gruber VA, Bradley JM, Bradley M, Delucchi K. A controlled trial of methadone treatment combined with directly observed isoniazid for tuberculosis prevention in injection drug users. Drug Alcohol Depend **2002**; 66:283-93.

3. Hirsch-Moverman Y, Colson PW, Bethel J, Franks J, El-Sadr WM. Can a peer-based intervention impact adherence to the treatment of latent tuberculous infection? Int J Tuberc Lung Dis **2013**; 17:1178-85.

4. Hovell M, Blumberg E, Gil-Trejo L, et al. Predictors of adherence to treatment for latent tuberculosis infection in high-risk Latino adolescents: a behavioral epidemiological analysis. Soc Sci Med **2003**; 56:1789-96.

5. Jasmer RM, Saukkonen JJ, Blumberg HM, et al. Short-course rifampin and pyrazinamide compared with isoniazid for latent tuberculosis infection: a multicenter clinical trial. Ann Intern Med **2002**; 137:640-7.

6. Jimenez-Fuentes MA, de Souza-Galvao ML, Mila Auge C, Solsona Peiro J, Altet-Gomez MN. Rifampicin plus isoniazid for the prevention of tuberculosis in an immigrant population. Int J Tuberc Lung Dis **2013**; 17:326-32.

7. Kominski GF, Varon SF, Morisky DE, et al. Costs and cost-effectiveness of adolescent compliance with treatment for latent tuberculosis infection: results from a randomized trial. J Adolesc Health **2007**; 40:61-8.

8. Malotte CK, Hollingshead JR, Larro M. Incentives vs outreach workers for latent tuberculosis treatment in drug users. Am J Prev Med **2001**; 20:103-7.

9. Nyamathi AM, Christiani A, Nahid P, Gregerson P, Leake B. A randomized controlled trial of two treatment programs for homeless adults with latent tuberculosis infection. Int J Tuberc Lung Dis **2006**; 10:775-82.

10. Trajman A, Long R, Zylberberg D, Dion MJ, Al-Otaibi B, Menzies D. Factors associated with treatment adherence in a randomised trial of latent tuberculosis infection treatment. Int J Tuberc Lung Dis **2010**; 14:551-9.

11. Tulsky JP, Hahn JA, Long HL, et al. Can the poor adhere? Incentives for adherence to TB prevention in homeless adults. Int J Tuberc Lung Dis **2004**; 8:83-91.

12. Berg J, Blumberg EJ, Sipan CL, et al. Somatic complaints and isoniazid (INH) side effects in Latino adolescents with latent tuberculosis infection (LTBI). Patient Educ Couns **2004**; 52:31-9.

13. Bock NN, Metzger BS, Tapia JR, Blumberg HM. A tuberculin screening and isoniazid preventive therapy program in an inner-city population. Am J Respir Crit Care Med **1999**; 159:295-300.

14. Fountain FF, Tolley E, Chrisman CR, Self TH. Isoniazid hepatotoxicity associated with treatment of latent tuberculosis infection: A 7-year evaluation from a public health tuberculosis clinic. Chest **2005**; 128:116-23.

15. Goswami ND, Gadkowski LB, Piedrahita C, et al. Predictors of latent tuberculosis treatment initiation and completion at a U.S. public health clinic: a prospective cohort study. BMC Public Health **2012**; 12:468.

16. Machado A, Jr., Finkmoore B, Emodi K, et al. Risk factors for failure to complete a course of latent tuberculosis infection treatment in Salvador, Brazil. Int J Tuberc Lung Dis **2009**; 13:719-25.

17. Martinez Sanchis A, Calpe Calpe JL, Llavador Ros G, Ena Munoz J, Calpe Armero A. [Primary prevention and treatment of latent tuberculosis infection with isoniazid: efficacy of a control program, 1997-2002]. Arch Bronconeumol **2005**; 41:27-33.

18. Minodier P, Lamarre V, Carle ME, Blais D, Ovetchkine P, Tapiero B. Evaluation of a school-based program for diagnosis and treatment of latent tuberculosis infection in immigrant children. J Infect Public Health **2010**; 3:67-75.

19. Morano JP, Walton MR, Zelenev A, Bruce RD, Altice FL. Latent tuberculosis infection: screening and treatment in an urban setting. J Community Health **2013**; 38:941-50.

20. Morisky DE, Ebin VJ, Malotte CK, Coly A, Kominski G. Assessment of tuberculosis treatment completion in an ethnically diverse population using two data sources. Implications for treatment interventions. Eval Health Prof **2003**; 26:43-58.

21. Narita M, Kellman M, Franchini DL, McMillan ME, Hollender ES, Ashkin D. Short-course rifamycin and pyrazinamide treatment for latent tuberculosis infection in patients with HIV infection: the 2-year experience of a comprehensive community-based program in Broward County, Florida. Chest **2002**; 122:1292-8.

22. Oni T, Tsekela R, Kwaza B, et al. A recent HIV diagnosis is associated with non-completion of Isoniazid Preventive Therapy in an HIV-infected cohort in Cape Town. PLoS One **2012**; 7:e52489.

23. Shieh FK, Snyder G, Horsburgh CR, Bernardo J, Murphy C, Saukkonen JJ. Predicting non-completion of treatment for latent tuberculous infection: a prospective survey. Am J Respir Crit Care Med **2006**; 174:717-21.

24. Shukla SJ, Warren DK, Woeltje KF, Gruber CA, Fraser VJ. Factors associated with the treatment of latent tuberculosis infection among health-care workers at a midwestern teaching hospital. Chest **2002**; 122:1609-14.

25. Trauer JM, Krause VL. Assessment and management of latent tuberculosis infection in a refugee population in the Northern Territory. Med J Aust **2011**; 194:579-82.

26. White MC, Tulsky JP, Menendez E, Goldenson J, Kawamura LM. Incidence of TB in inmates with latent TB infection: 5-year follow-up. Am J Prev Med **2005**; 29:295-301.

27. Young J, Edick T, Klee D, O'Connor ME. Successful treatment of pediatric latent tuberculosis infection in a community health center clinic. Pediatr Infect Dis J **2012**; 31:e147-51.

28. Ailinger RL, Dear MR. Adherence to tuberculosis preventive therapy among Latino immigrants. Public Health Nurs **1998**; 15:19-24.

29. Ailinger RL, Black P, Nguyen N, Lasus H. Predictors of Adherence to Latent Tuberculosis Infection Therapy in Latino Immigrants. Journal of Community Health Nursing **2007**; 24:191-8.

30. Anger HA, Proops D, Harris TG, et al. Active case finding and prevention of tuberculosis among a cohort of contacts exposed to infectious tuberculosis cases in New York City. Clin Infect Dis **2012**; 54:1287-95.

31. Anibarro L, Casas S, Paz-Esquete J, et al. Treatment completion in latent tuberculosis infection at specialist tuberculosis units in Spain. Int J Tuberc Lung Dis **2010**; 14:701-7.

32. Cass AD, Talavera GA, Gresham LS, Moser KS, Joy W. Structured behavioral intervention to increase children's adherence to treatment for latent tuberculosis infection. Int J Tuberc Lung Dis **2005**; 9:415-20.

33. Chang AH, Polesky A, Bhatia G. House calls by community health workers and public health nurses to improve adherence to isoniazid monotherapy for latent tuberculosis infection: a retrospective study. BMC Public Health **2013**; 13:894.

34. Chang SH, Eitzman SR, Nahid P, Finelli ML. Factors associated with failure to complete isoniazid therapy for latent tuberculosis infection in children and adolescents. J Infect Public Health **2014**; 7:145-52.

35. Codecasa LR, Murgia N, Ferrarese M, et al. Isoniazid preventive treatment: predictors of adverse events and treatment completion. Int J Tuberc Lung Dis **2013**; 17:903-8.

36. Cruz AT, Starke JR. Increasing adherence for latent tuberculosis infection therapy with health department-administered therapy. Pediatr Infect Dis J **2012**; 31:193-5.

37. Fresard I, Bridevaux PO, Rochat T, Janssens JP. Adverse effects and adherence to treatment of rifampicin 4 months vs isoniazid 6 months for latent tuberculosis: a retrospective analysis. Swiss Med Wkly **2011**; 141:w13240.

38. Gershon AS, McGeer A, Bayoumi AM, Raboud J, Yang J. Health care workers and the initiation of treatment for latent tuberculosis infection. Clin Infect Dis **2004**; 39:667-72.

39. Gilroy SA, Rogers MA, Blair DC. Treatment of latent tuberculosis infection in patients aged > or =35 years. Clin Infect Dis **2000**; 31:826-9.

40. Grinsdale JA, Ho CS, Banouvong H, Kawamura LM. Programmatic impact of using QuantiFERON(R)-TB Gold in routine contact investigation activities. Int J Tuberc Lung Dis **2011**; 15:1614-20.

41. Haley CA, Stephan S, Vossel LF, Sherfy EA, Laserson KF, Kainer MA. Successful use of rifampicin for Hispanic foreign-born patients with latent tuberculosis infection. Int J Tuberc Lung Dis **2008**; 12:160-7.

42. Hirsch-Moverman Y, Bethel J, Colson PW, Franks J, El-Sadr W. Predictors of latent tuberculosis infection treatment completion in the United States: an inner city experience. Int J Tuberc Lung Dis **2010**; 14:1104-11.

43. Horsburgh CR, Jr., Goldberg S, Bethel J, et al. Latent TB infection treatment acceptance and completion in the United States and Canada. Chest **2010**; 137:401-9.

44. Kan B, Kalin M, Bruchfeld J. Completing treatment for latent tuberculosis: patient background matters. Int J Tuberc Lung Dis **2013**; 17:597-602.

45. Kwara A, Herold JS, Machan JT, Carter EJ. Factors associated with failure to complete isoniazid treatment for latent tuberculosis infection in Rhode Island. Chest **2008**; 133:862-8.

46. Lardizabal A, Passannante M, Kojakali F, Hayden C, Reichman LB. Enhancement of treatment completion for latent tuberculosis infection with 4 months of rifampin. Chest **2006**; 130:1712-7.

47. Lee AM, Mennone JZ, Jones RC, Paul WS. Risk factors for hepatotoxicity associated with rifampin and pyrazinamide for the treatment of latent tuberculosis infection: experience from three public health tuberculosis clinics. Int J Tuberc Lung Dis **2002**; 6:995-1000.

48. Li J, Munsiff SS, Tarantino T, Dorsinville M. Adherence to treatment of latent tuberculosis infection in a clinical population in New York City. Int J Infect Dis **2010**; 14:e292-7.

49. Lobato MN, Reves RR, Jasmer RM, Grabau JC, Bock NN, Shang N. Adverse events and treatment completion for latent tuberculosis in jail inmates and homeless persons. Chest **2005**; 127:1296-303.

50. LoBue PA, Moser KS. Use of isoniazid for latent tuberculosis infection in a public health clinic. Am J Respir Crit Care Med **2003**; 168:443-7.

51. Lopez G, Wood M, Ayesta FJ. [10 years of innovation in the treatment of latent tuberculosis infection: a comparison between standard and short course therapies in directly observed therapy]. Rev Esp Sanid Penit **2011**; 13:3-14.

52. Marks SM, Taylor Z, Qualls NL, Shrestha-Kuwahara RJ, Wilce MA, Nguyen CH. Outcomes of contact investigations of infectious tuberculosis patients. Am J Respir Crit Care Med **2000**; 162:2033-8.

53. McElroy PD, Ijaz K, Lambert LA, et al. National survey to measure rates of liver injury, hospitalization, and death associated with rifampin and pyrazinamide for latent tuberculosis infection. Clin Infect Dis **2005**; 41:1125-33.

54. Mindachew M, Deribew A, Tessema F, Biadgilign S. Predictors of adherence to isoniazid preventive therapy among HIV positive adults in Addis Ababa, Ethiopia. BMC Public Health **2011**; 11:916.

55. Nuzzo JB, Golub JE, Chaulk P, Shah M. Analysis of Latent Tuberculosis Infection Treatment Adherence Among Refugees and Other Patient Groups Referred to the Baltimore City Health Department TB Clinic, February 2009-March 2011. J Immigr Minor Health **2013**.

56. Page KR, Sifakis F, Montes de Oca R, et al. Improved adherence and less toxicity with rifampin vs isoniazid for treatment of latent tuberculosis: a retrospective study. Arch Intern Med **2006**; 166:1863-70.

57. Parsyan AE, Saukkonen J, Barry MA, Sharnprapai S, Horsburgh CR, Jr. Predictors of failure to complete treatment for latent tuberculosis infection. J Infect **2007**; 54:262-6.

58. Priest DH, Vossel LF, Jr., Sherfy EA, Hoy DP, Haley CA. Use of intermittent rifampin and pyrazinamide therapy for latent tuberculosis infection in a targeted tuberculin testing program. Clin Infect Dis **2004**; 39:1764-71.

59. Rennie TW, Bothamley GH, Engova D, Bates IP. Patient choice promotes adherence in preventive treatment for latent tuberculosis. Eur Respir J **2007**; 30:728-35.

60. Vinnard C, Gopal A, Linkin DR, Maslow J. Isoniazid Toxicity among an Older Veteran Population: A Retrospective Cohort Study. Tuberc Res Treat **2013**; 2013:549473.

61. White MC, Gournis E, Kawamura M, Menendez E, Tulsky JP. Effect of directly observed preventive therapy for latent tuberculosis infection in San Francisco. Int J Tuberc Lung Dis **2003**; 7:30-5.

62. Young H, Wessolossky M, Ellis J, Kaminski M, Daly JS. A retrospective evaluation of completion rates, total cost, and adverse effects for treatment of latent tuberculosis infection in a public health clinic in central massachusetts. Clin Infect Dis **2009**; 49:424-7.

63. Menzies D, Dion MJ, Rabinovitch B, Mannix S, Brassard P, Schwartzman K. Treatment completion and costs of a randomized trial of rifampin for 4 months versus isoniazid for 9 months. Am J Respir Crit Care Med **2004**; 170:445-9.

64. Menzies D, Long R, Trajman A, et al. Adverse events with 4 months of rifampin therapy or 9 months of isoniazid therapy for latent tuberculosis infection: a randomized trial. Ann Intern Med **2008**; 149:689-97.

65. Spyridis NP, Spyridis PG, Gelesme A, et al. The effectiveness of a 9-month regimen of isoniazid alone versus 3- and 4-month regimens of isoniazid plus rifampin for treatment of latent tuberculosis infection in children: results of an 11-year randomized study. Clin Infect Dis **2007**; 45:715-22.

66. Sterling TR, Villarino ME, Borisov AS, et al. Three months of rifapentine and isoniazid for latent tuberculosis infection. N Engl J Med **2011**; 365:2155-66.

67. Tortajada C, Martinez-Lacasa J, Sanchez F, et al. Is the combination of pyrazinamide plus rifampicin safe for treating latent tuberculosis infection in persons not infected by the human immunodeficiency virus? Int J Tuberc Lung Dis **2005**; 9:276-81.

68. Chaisson RE, Barnes GL, Hackman J, et al. A randomized, controlled trial of interventions to improve adherence to isoniazid therapy to prevent tuberculosis in injection drug users. Am J Med **2001**; 110:610-5.

69. Matteelli A, Casalini C, Raviglione MC, et al. Supervised preventive therapy for latent tuberculosis infection in illegal immigrants in Italy. Am J Respir Crit Care Med **2000**; 162:1653-5.

70. White MC, Tulsky JP, Goldenson J, Portillo CJ, Kawamura M, Menendez E. Randomized controlled trial of interventions to improve follow-up for latent tuberculosis infection after release from jail. Arch Intern Med **2002**; 162:1044-50.

71. Hovell MF, Sipan CL, Blumberg EJ, et al. Increasing Latino adolescents' adherence to treatment for latent tuberculosis infection: a controlled trial. Am J Public Health **2003**; 93:1871-7.

72. Ailinger RL, Martyn D, Lasus H, Lima Garcia N. The effect of a cultural intervention on adherence to latent tuberculosis infection therapy in Latino immigrants. Public Health Nurs **2010**; 27:115-20.

73. Goldberg SV, Wallace J, Jackson JC, Chaulk CP, Nolan CM. Cultural case management of latent tuberculosis infection. Int J Tuberc Lung Dis **2004**; 8:76-82.

74. Sahni R, Miranda C, Yen-Lieberman B, et al. Does the implementation of an interferon-gamma release assay in lieu of a tuberculin skin test increase acceptance of preventive therapy for latent tuberculosis among healthcare workers? Infect Control Hosp Epidemiol **2009**; 30:197-9.
